# Expanding structural insights into DNA packaging apparatus and endolysin LysSA05 function of Epsilon15 bacteriophage

**DOI:** 10.3389/fcimb.2025.1643576

**Published:** 2025-08-14

**Authors:** Muhammad Saleem Iqbal Khan, Ju Wu, Shenlin Ji, Demeng Tan, Bingrui Sui, Shanshan Peng, Jinbiao Zhan, Jiajun Yin

**Affiliations:** ^1^ Department of General Surgery, Affiliated Zhongshan Hospital of Dalian University, Dalian, Liaoning, China; ^2^ Shanghai Public Health Clinical Center, Fudan University, Shanghai, China; ^3^ MOE Key Laboratory of Bio-Intelligent Manufacturing, School of Bioengineering, Dalian University of Technology, Dalian, China; ^4^ Department of Biochemistry, The Second Affiliated Hospital, School of Medicine, Zhejiang University, Hangzhou, China

**Keywords:** bacteriophage, electron microscopy, cryo-electron microscopy, endolysin, antimicrobial peptides, multidrug-resistant bacteria

## Abstract

The rising prevalence of multidrug-resistant (MDR) foodborne pathogens, particularly *Salmonella* spp., necessitates alternative antimicrobial solutions. Phage therapy offers a promising solution against MDR Gram-negative infections; however, its clinical application is constrained by the presence of endotoxins, residual cellular debris, the risk of horizontal gene transfer by temperate phages, and an incomplete understanding of how phage structural integrity influences infectivity and enzyme function. In this study, we present a structural and functional analysis of temperate bacteriophage Epsilon15 (ϵ15), focusing on its DNA packaging and injection machinery, along with characterization of the dual-acting endolysin LysSA05. Iodixanol-purified virions suspended in phosphate-buffered saline (PBS), under conditions optimized to preserve virion stability, were analyzed using graphene oxide (GO)-supported cryo-electron microscopy. This approach resolved the full asymmetric architecture of ϵ15, revealing a detailed internal nucleic acid organization with at least eight concentric layers radially and approximately 28 axially compacted layers within the capsid. The DNA packaging machinery, comprising the core, portal, and hub, was resolved at high resolution, including a 42 nm-long and 18 nm-wide injection channel anchored by a dodecameric portal complex visualized at ~7 Å resolution. Concurrently, we characterized LysSA05, a dual-acting endolysin harboring a glycoside hydrolase 19 (GH19) catalytic domain accommodating peptidoglycan (PG) residues *N*-acetylmuramic acid (NAM) and *N*-acetylglucosamine (NAG) through structural docking, indicating plausible binding interactions that promote hydrolysis support vector machine (SVM), random forest (RF), discriminant analysis (DA), artificial neural network (ANN) and physicochemical scanning identified an amphipathic helix (residues 59-112) with predicted antimicrobial peptide (AMP)-like properties. Biochemical validation confirmed that LysSA05 destabilizes lipopolysaccharides (LPS) and permeabilizes the outer membrane of Gram-negative bacteria independently of permeabilizers, with enhanced efficacy observed upon co-treatment with Ethylenediaminetetraacetic acid (EDTA) or citric acid. In summary, our findings elucidate key structural features of ϵ15 relevant to infection and genome delivery, while positioning LysSA05 as a promising enzybiotic candidate against MDR Gram-negative pathogens.

## 1 Introduction

Members of the *Enterobacteriaceae* family, such as *Salmonella* spp. and *Escherichia coli* species, are Gram-negative pathogens responsible for a substantial burden of food-borne and gastrointestinal infections in both humans and animals (Authority et al., 2020). According to the Centers for Disease Control and Prevention (CDC), non-typhoidal *Salmonella* infections affect approximately 1.35 million individuals annually in the United States alone (Daigle, 2021). The rapid emergence and dissemination of multidrug-resistant (MDR) pathogens have intensified efforts to identify alternative antimicrobial strategies (Medalla et al., 2021; Tadesse et al., 2012).

Bacteriophages, as natural predators of bacteria, have gained renewed attention as targeted biocontrol agents, due to their specificity and lytic efficacy (Jayamanne and Foddai, 2025; Lin et al., 2018). However, their clinical use is limited by safety concerns, regulatory hurdles, and gaps in understanding phage biology and structure-function relationships. Among these, bacteriophage Epsilon15 (ϵ15) is a well-characterized, tailed temperate double-stranded DNA (dsDNA) phage that infects *S. enterica* serovar Anatum, a Gram-negative bacterium responsible for gastrointestinal infections in humans and animals (Kropinski et al., 2007). It exhibits host specificity via endorhamnosidase-mediated recognition of the LPS-O antigen, causing the tail hub to open and the core to exit the capsid and form a tube across the periplasmic space to introduce viral DNA into a cytoplasm of the host cell. These features make ϵ15 a valuable model for understanding phage-host interactions and for potential applications in food safety (Chang et al., 2010). Beyond their lytic activity, temperate phages also drive horizontal gene transfer, contributing to bacterial evolution and dissemination of antibiotic-resistance genes (Penadés et al., 2025).

Central to ϵ15’s infection cycle is a precisely coordinated system involving an icosahedral capsid, a tail for host attachment, and a sophisticated DNA packaging motor that ensures accurate genome translocation (Chang et al., 2010). The structure of its tail DNA injection apparatus enables a better understanding of the mechanism by which bacteriophages cause genetic variation in their host. While early cryo-electron microscopy (EM) studies revealed ϵ15 asymmetric architecture, including a dodecameric portal complex and coaxially layered DNA was resolved at 20 Å (Jiang et al., 2006). Later, symmetry-imposed reconstructions resolved the capsid shell at near-atomic resolution and identified major capsid protein gp7 and minor capsid protein gp10 (Baker et al., 2013), while cryo-EM captured early infection stages and tail spike activity in *Salmonella* (Chang et al., 2010). According to genomic analysis, ϵ15 tail nucleic acid packaging/injection device is mainly composed of portal protein, corresponding to gp4, gp20, gp15 and gp17/16, respectively (Guichard et al., 2013). However, limitations in purification such as cellular debris, endotoxins and physicochemical stress, have hindered detailed analysis of infectious phage particles and constrained their potential as therapeutic agents (Reindel and Fiore, 2017; Jończyk-Matysiak et al., 2019).

Bacteriophages use precisely coordinated structures to ensure replication fidelity and to control the spatial activation of lytic enzymes such as endolysins during host infection. Endolysins are peptidoglycan (PG)-degrading enzymes that act at the end of the phage replication cycle, cleaving glycosidic bonds between *N*-acetylmuramic acid (NAM) and *N*-acetylglucosamine (NAG) (Fernandes and São-José, 2018). In Gram-negative bacteria, endolysins are typically small (~15-20 kDa) with single or dual catalytic domains, such as glycosidase, amidase, or peptidase (Nazir et al., 2023; Wang et al., 2023). Their activity is often limited by the outer membrane but can be enhanced by membrane-permeabilizing agents or fusion with antimicrobial peptide (AMP)-like domains, such as Artilysins (Islam et al., 2022). Some broad-spectrum lysins, like LysO78, Abtn-4, Ply6A3 LysAB2 and AbEndolysin, show strong activity against Gram-negative pathogens (Deng et al., 2021; Lai et al., 2011; Wu et al., 2019; Yuan et al., 2021). Most of these endolysins exhibit antimicrobial potential due to a predicted C-terminal amphipathic α-helical domain with AMP-like properties (Kim et al., 2025b; Yuan et al., 2021; Peng et al., 2017; Wu et al., 2024).

In this study, we integrate high-resolution structural biology with bioinformatic and machine learning-driven functional enzymology to advance phage-based antimicrobial strategies. By employing iodixanol gradient purification and graphene oxide (GO)-supported vitrification, we preserved Epsilon (ϵ15) in a near-native, infectious state, enabling asymmetric reconstruction of the full virion at ~7 Å resolution. This reconstruction revealed detailed features of the internal nucleic acid and DNA packaging machinery, including the core, portal, and hub regions, with a portal complex exhibiting dodecameric symmetry and forming a 42 nm-long, 18 nm-wide packaging channel. Simultaneously, we identified an amphipathic helix (residues 59-112) in LysSA05, predicted via machine learning to possess AMP-like properties, and biochemically validated its ability to destabilize *Salmonella* and *E. coli* LPS. LysSA05’s natural fusion of a glycoside hydrolase 19 (GH19) PG-hydrolase and outer membrane-permeabilizing AMP domain enables adjuvant-free lytic activity against MDR clinical isolates. The results indicate that ϵ15 is a valuable model system for understanding phage architecture and lytic enzyme function, offering a promising foundation for the development of enzybiotic-based therapeutics against Gram-negative pathogens.

## 2 Materials and methods

### 2.1 Bacterial strains, phage, and growth conditions

The bacterial strains, phage, and plasmid used in this study are listed in **Supplementary Table 1**. Bacteriophage ϵ15 was propagated using *S. enterica* serovar Anatum in 2× Yeast Extract-Tryptone (YT) broth at 37°C. Phage titers were determined using the plaque-forming unit (PFU) method and stored in phosphate-buffered saline (PBS) at 4°C for subsequent analysis. For cloning and protein expression, *E. coli* strains DH5α, BL21 (DE3), and Rosetta (DE3) were cultured in Luria Bertani (LB) medium (per liter: 10 g tryptone, 5 g yeast extract, 5 g NaCl) supplemented with kanamycin (100 μg/mL). The LysSA05 endolysin gene was cloned into the pET-28a(+) plasmid, incorporating a C-terminal 6×His-tag for affinity purification. *S. anatum* and *E. coli* C were used as model Gram-negative strains for LPS extraction and lytic activity assays. Additional MDR clinical isolates of *Salmonella* and *E. coli* were tested to evaluate the antibacterial spectrum (**Supplementary Table 2**). All bacterial strains were grown in LB broth or agar at 37°C with shaking at 220 rpm. Bacterial viability was assessed by colony-forming unit (CFU) per mL, enumeration under sterile conditions.

### 2.2 Propagation and purification of bacteriophage ϵ15

Bacteriophage ϵ15 was propagated using *S. anatum* as the host. An overnight culture of *S. anatum* was diluted 1:100 in 5 mL of 2 × YT broth and incubated at 37°C until reaching an OD_600_ of 0.4. Phage suspension (1 × 10^9^ PFU/mL) and 20% sterile-filtered glucose were added to facilitate adsorption, followed by a 4h incubation. The culture was centrifuged at 7,155 × g for 15 min at 4°C, and the pellet was resuspended in 250 mL of 2× YT broth. Incubation continued at 37°C for 16 h until complete lysis was observed. The lysate was clarified by centrifugation at 7,155 × g for 15 min at 4°C to remove cellular debris. Phage particles were precipitated by adding polyethylene glycol (PEG) 8000 to a final concentration of 20% and NaCl to 1.5 M, followed by incubation at 4°C for 4 h. SM buffer was prepared by dissolving 11.7 g NaCl, 1.4 g MgSO₄, and 50 mL of 1 M Tris-HCl (pH 7.5) in 950 mL of demineralized water. For SM-gelatin buffer, 2% (w/v) gelatin was added to the SM buffer. The phage solution was kept at 4°C for subsequent analysis. Bacteriophage ϵ15 particles were initially concentrated by ultracentrifugation over a 12% sucrose cushion (105,000 × g, 2 h, 4°C), and the resulting pellet was resuspended in 300 µL of PBS and incubated overnight at 4°C. For further purification, samples were subjected to various density gradients, including discontinuous and continuous sucrose gradients, as well as a discontinuous iodixanol gradient (10-55%, Sigma Aldrich, D1556). The final preparation for cryo-electron microscopy (cryo-EM) was obtained using a discontinuous iodixanol gradient. Gradients were formed as described previously (Khan et al., 2021) with layers of 10%, 20%, 30%, 40%, and 55% iodixanol and centrifuged in an SW41 rotor at 175,000 × g for 4 h at 4°C. A distinct white band was collected, washed using 14 kDa MWCO (Amicon^®^), and resuspended in PBS. The final phage titer was 1.5 × 10^10^ PFU/mL, determined by standard plaque assay (Sui et al., 2025), and stored at 4°C until negative staining and cryo-EM imaging.

### 2.3 Negative staining and transmission electron microscopy

For morphological and purity analysis, ϵ15 phage particles were subjected to negative staining and observed under transmission electron microscopy (TEM). Carbon-coated copper grids were glow discharged at 15 mA for 60 s, using a PELCO easiGlow™ 91000 system (USA) to render them hydrophilic. A 3 µL aliquot of 10-fold diluted phage sample was applied to each grid and allowed to adsorb for 30 s. Excess liquid was blotted with filter paper, and the grid was stained with 2% aqueous uranyl acetate (UA) in three intervals: two brief applications of 10 s each, followed by a final application lasting 1 min. Grids were air-dried at room temperature for at least 1 min before imaging. Micrographs were acquired at magnifications of 87kx and 105kx using a FEI Tecnai Spirit 120 kV microscope at the Center for EM, Zhejiang University School of Medicine. After confirming the morphology, integrity, and purity of the sample, cryo-EM vitrification was performed.

### 2.4 Cryo-EM grid preparation

Cryo-EM samples were prepared using two types of grids: graphene oxide (GO; Sigma Aldrich; 763705) coated and uncoated Quantifoil R1.2/1.3 Cu 300 mesh grids. For GO coating, grids were glow discharged at 25 mA for 180 s. GO (2 mg/mL) was diluted to 0.4 mg/mL, and 2.5 µL was applied to each grid. After 180 s incubation period, excess solution was removed, and the grids were washed twice with 10 µL ultrapure water, followed by a 5 min air-drying. For both grid types, 2.5 µL of purified ϵ15 phage sample were applied. Using the Vitrobot (FEI) set at 100% humidity and 22°C, grids was incubated with sample for 60 s, blotted for 6.5 s, and then rapidly plunged into liquid ethane. Cryo-EM imaging was performed using a Thermo Fisher Talos F200C microscope after confirming grid quality. The second grid type underwent direct hydrophilic treatment and sample application without GO coating.

### 2.5 Cryo-EM data collection and processing

High-resolution data were collected using a Thermo Fisher FEI Titan Krios cryo-EM operating at 300 kV. Microscope calibration included coma-free alignment and beam-tilt minimization. Data acquisition was conducted using SerialEM software (Schorb et al., 2019). Images were recorded in super-resolution mode on a Gatan K2 Summit direct electron detector at a nominal magnification of 22,500×, with a pixel size of 1.307 Å/pixel after binning. The total electron dose was ~50 e^-^/Å^2^, with an exposure time of 10 s and a frame rate of 4 frames per second. The defocus range was set between -2.0 µm and -3.0 µm. Drift correction was performed using MotionCor2 (Zheng et al., 2017), producing aligned and dose-weighted images for downstream analysis. Automated particle picking was conducted using Gautomatch (https://www.mrc-lmb.cam.ac.uk/kzhang/Gautomatch/). Gctf was used to estimate local defocus and correct astigmatism. Image processing, including 2D classification, 3D classification, and 3D reconstruction, was performed using RELION 2.0 (Scheres, 2016). The resulting cryo-electron density map provided structural insights into the ϵ15 virion.

### 2.6 Bioinformatics analysis

The endolysin gene *LysSA05* (locus tag: epsilon15_*gp05*, accession number: NP_848233.1) was analyzed using a comprehensive bioinformatics pipeline. Sequence identity and function were assessed via BLAST, PSI-BLAST, HMMER (Potter et al., 2018), and InterProScan (Blum et al., 2025). Domain architecture was predicted with HHpred (Söding et al., 2005), and physicochemical properties including molecular weight (MW) and isoelectric point (pI) were computed using ProtParam (Gasteiger et al., 2005). Multiple sequence alignment with homologs, including *Salmonella* phage SPN1S, was performed using Clustal Omega (Sievers and Higgins, 2018), and visualized in ESPript 3.0 (Robert and Gouet, 2014). Additional homologs identified via HMMER were aligned using MUSCLE (https://www.ebi.ac.uk/jdispatcher/msa) and used to construct a phylogenetic tree in MEGA X (Kumar et al., 2024). Secondary structure was predicted using PSIPRED (Jones, 1999) and PHD (https://npsa-prabi.ibcp.fr/cgi-bin/npsa_automat.pl?page=/NPSA/npsa_phd.html), while 3D modeling was conducted with Phyre2 (Kelley et al., 2015). The model was validated using ERRAT and PROCHECK through the SAVES server, and structural pockets were analyzed using Phyre2 “Investigator” module. Molecular docking was performed using CB-Dock2 (Liu et al., 2020), targeting PG residues NAM (SID: 24890714) and NAG (CID: 1738118). A homology model of LysSA05 was also generated using Swiss-Model. Docking simulations employed AutoDock Vina with automated cavity detection. Top-ranked poses were selected based on binding affinity (kcal/mol) and cavity volume and were visualized in 3D (within CB-Dock) and 2D formats within LigPlot+ v.2.2 (Wallace et al., 1995).

### 2.7 Physicochemical profiling and AMP prediction

The full-length LysSA05 protein (208 amino acids) was computationally segmented into overlapping peptides of 54 residues (pre-screening) and 20 residues (post-screening) for AMP prediction. A complementary 54-residues sliding window (three α-helical turns) was applied using HeliQuest (https://heliquest.ipmc.cnrs.fr/index.html) to assess amphipathic helicity (Gautier et al., 2008). For each segment, net charge (z), hydrophobic moment (µH), and average hydrophobicity (H) were calculated. Peptides with z ≥ 2, µH ≥ 0.4, and amphipathic features were shortlisted and ranked based on combined physicochemical scores. Helical wheel diagrams visualized residue distribution. Subsequently, 189 overlapping 20-mer peptides were submitted to the CAMP3 server (http://www.camp3.bicnirrh.res.in/) for AMP and non-AMP (NAMP) classification (Thomas et al., 2010) and peptides were evaluated using four classifiers: support vector machine (SVM), random forest (RF), discriminant analysis (DA), and artificial neural network (ANN). Prediction outputs were visualized via violin plots, boxplots, sequence position heatmaps, and Venn diagrams. Finally, the overlapping peptide in machine learning (ML) classifiers and HeliQuest were selected and these peptide underwent *de novo* 3D modeling using PEP-FOLD4 (https://bioserv.rpbs.univ-paris-diderot.fr/services/PEP-FOLD4/) under physiological conditions (pH 7.0, 150 mM ionic strength), validating the presence of membrane-active amphipathic helices (Rey et al., 2023).

### 2.8 Genomic DNA extraction, cloning, and expression of LysSA05 endolysin

Genomic DNA was extracted from purified ϵ15 phage particles using the M13 DNA Extraction Kit (Takara) and further purified by agarose gel electrophoresis and gel extraction (Omega Bio-Tek). DNA concentration and purity were assessed using a NanoDrop spectrophotometer. The *LysSA05* gene (*gp05*, 628 bp) was amplified by PCR using primers designed in SnapGene and incorporating *NcoI* and *XhoI* restriction sites: Forward: 5′-CATGCCATGGACATTAACCAGTTCCGGCGC-3′, Reverse: 5′-CCGCTCGAGTACCGCCAGCACCTTACTGGC-3′. PCR was performed using 2× Hieff Master Mix (Yeasen), and the amplicon was purified and digested with *NcoI* and *XhoI*. The PCR product was ligated into the expression vector pET-28a(+) using T4 DNA ligase, to generate the recombinant plasmid pET-28a(+)/*LysSA05*. The construct was transformed into *E. coli* DH5α via CaCl_2_ heat-shock transformation, and positive clones were screened by colony PCR and confirmed by Sanger sequencing. For protein expression, verified plasmids were introduced into *E. coli* BL21 (DE3) and Rosetta (DE3) strains. Cultures were grown in LB medium supplemented with kanamycin (100 μg/mL) to mid-log phase (OD_600_ = 0.4) and induced with Isopropyl β-D-1-thiogalactopyranoside (IPTG) at varying IPTG concentrations (0.1, 0.5, 1.0 mM) at either 30°C or 37°C for 5 h. Cells were harvested, lysed by sonication, and the soluble and insoluble fractions were analyzed by SDS-PAGE.

### 2.9 Purification, refolding, and SDS-PAGE analysis of recombinant LysSA05

Recombinant LysSA05 was predominantly expressed as insoluble inclusion bodies in both *E. coli* BL21 and Rosetta host strains. Harvested cells were lysed by sonication and centrifuged at 16,099 × g for 10 min. Inclusion bodies were washed with lysis buffer (PBS + 0.1% Triton X-100) and ddH_2_O, then solubilized in Nitrilotriacetic Acid (NTA) buffer containing 8 M urea and purified via Ni^2+^-NTA resin under denaturing conditions. The column was pre-equilibrated with binding buffer prior to sample loading. LysSA05 was eluted with a gradient of imidazole (20–250 mM) in 1× NTA buffer (8 M urea), and eluted fractions were analyzed by SDS-PAGE (12% gel) using the Laemmli protocol (sample denaturation at 95°C for 5 min in SDS loading buffer) (He, 2011). Gels were stained with Coomassie Brilliant Blue R250 for 1 h and de-stained using a methanol: acetic acid: water solution (3:1:6, v/v/v) until a clear background was achieved. A distinct ~25 kDa band corresponding to His-tagged LysSA05 was used to evaluate expression levels, solubility, and purification efficiency. Fractions containing > 90% pure LysSA05 were pooled and subjected to stepwise refolding by dialysis. The protein was first dialyzed against decreasing concentrations of urea (8 M, 4 M, 2 M, 0 M) in NTA buffer at 4°C. This was followed by sequential dialysis against: Buffer A: 20 mM Tris-Cl, 0.5 mM NaCl, 5% glycerol, 5% sucrose, 1% arginine, pH 8.0, Buffer B: 20 mM Tris-Cl, 0.5 mM NaCl, pH 8.0, Buffer C: 1× PBS supplemented with 5% glycerol, 5% sucrose, and 1% arginine, pH 7.4. Each dialysis step was carried out for 2 h at 4°C under gentle agitation. The final refolded protein was dialyzed in 1× PBS, concentrated using PEG 20,000, and filtered (0.22 μm). Protein concentration was measured using Bicinchoninic Acid (BCA) and stored at 4°C for downstream assays.

### 2.10 Lipopolysaccharides, silver staining, and degradation assessment

LPS was extracted from *E. coli* C and *S. anatum* using a modified hot phenol water method (Davis Jr and Goldberg, 2012). Briefly, mid-log phase cultures were harvested, washed with PBS containing 0.15 mM CaCl_2_ and 0.5 mM MgCl_2_, and lysed by sonication. Lysates were sequentially treated with proteinase K, DNase I, and RNase A, followed by phenol extraction at 65-70°C. LPS was precipitated with 0.5 M sodium acetate and 95% ethanol at -20°C overnight, then collected by centrifugation. Samples were extensively dialyzed against distilled water, lyophilized, and stored at 4°C. To assess the LPS degrading activity of LysSA05, 50 µL of purified LPS from *E. coli* C or *S. anatum* was incubated with recombinant LysSA05 (100 μg/mL) at 37°C for 6 h. Degradation was evaluated by SDS-PAGE followed by Ni^2+^-enhanced silver staining and Coomassie Brilliant Blue staining for protein control. By applying this method, we could monitor the enzymatic activity of LysSA05 on LPS substrates, as degradation would result in altered banding patterns on the gel. As previously reported, silver staining has been used to visualize the LPS from Gram-negative bacteria (Tan and Grewal, 2002). No outer membrane permeabilizers were used, enabling direct assessment of LysSA05 activity on intact LPS. During silver staining, LPS was visualized using the Sangon Biotech Silver Staining Kit (C500029) according to the manufacturer’s instructions. Gels were treated with solutions A-E, with protein and LPS bands appearing as dark signals. The reaction was stopped with solution E, and gels were rinsed with ddH_2_O. LPS purity was confirmed by comparing silver-stained and Coomassie-stained SDS-PAGE gels.

### 2.11 Assessment of LysSA05 lytic activity

The dual lytic activity of LysSA05 was assessed *in vitro* against *S. anatum* and *E. coli* C to evaluate its ability to degrade both LPS and PG. Bacterial cultures were grown overnight in LB broth at 37°C, harvested at OD_600_ = 0.4, washed with PBS (pH 7.4), and resuspended to a final concentration of 10^8^ CFU/mL. Purified LysSA05 (100 μg/mL) was added to 800 μL of bacterial suspension and incubated at 37°C for 5 h. Negative controls received an equal volume of PBS without enzyme. To investigate the impact of outer membrane permeabilizers (OMPs), additional treatments were performed with LysSA05 in the presence of Ethylenediaminetetraacetic acid (EDTA) or citric acid (0-0.5 mM) for 1 min at 37°C. Antibacterial activity was quantified by CFU enumeration, and results were expressed as reductions in viable bacterial counts. All experiments were conducted in triplicate to ensure reproducibility.

### 2.12 Antibacterial spectrum of different food-borne isolates

After confirming the lytic activity of endolysin LysSA05, its antibacterial efficacy was further evaluated against MDR clinical isolates of *Salmonella* and *E. coli* (**Supplementary Table S2**). The susceptibility of these resistant isoltes to LysSA05 (100 μg/mL) was analyzed using a CFU/mL plating assay. Prior to treatment, the clinical isolates were cultured in LB medium to an optical density (OD_600_) of 0.4 and incubated at 37°C for 1 h. Following endolysin treatment, bacterial viability was assessed by CFU/mL enumeration. All experiments were conducted in triplicate to ensure accuracy and reproducibility.

### 2.13 Statistical analysis

Statistical analyses and graph generation were conducted using GraphPad Prism software (GraphPad Prism 9.2, La Jolla, CA). Data are presented as the mean ± standard deviation (SD). Statistical significance was assessed using one-way ANOVA followed by Turkey’s multiple comparison test to compare stability and infectivity of the phages in the presence of the various buffers and gradient techniques. A *P*-value < 0.05 was considered statistically significant; *P ≤ 0.05; **P ≤ 0.01; ***P ≤ 0.001; and ****P ≤ 0.0001.

## 3 Results

### 3.1 Assessing stability and morphological integrity of ϵ15

**Figure 1:**
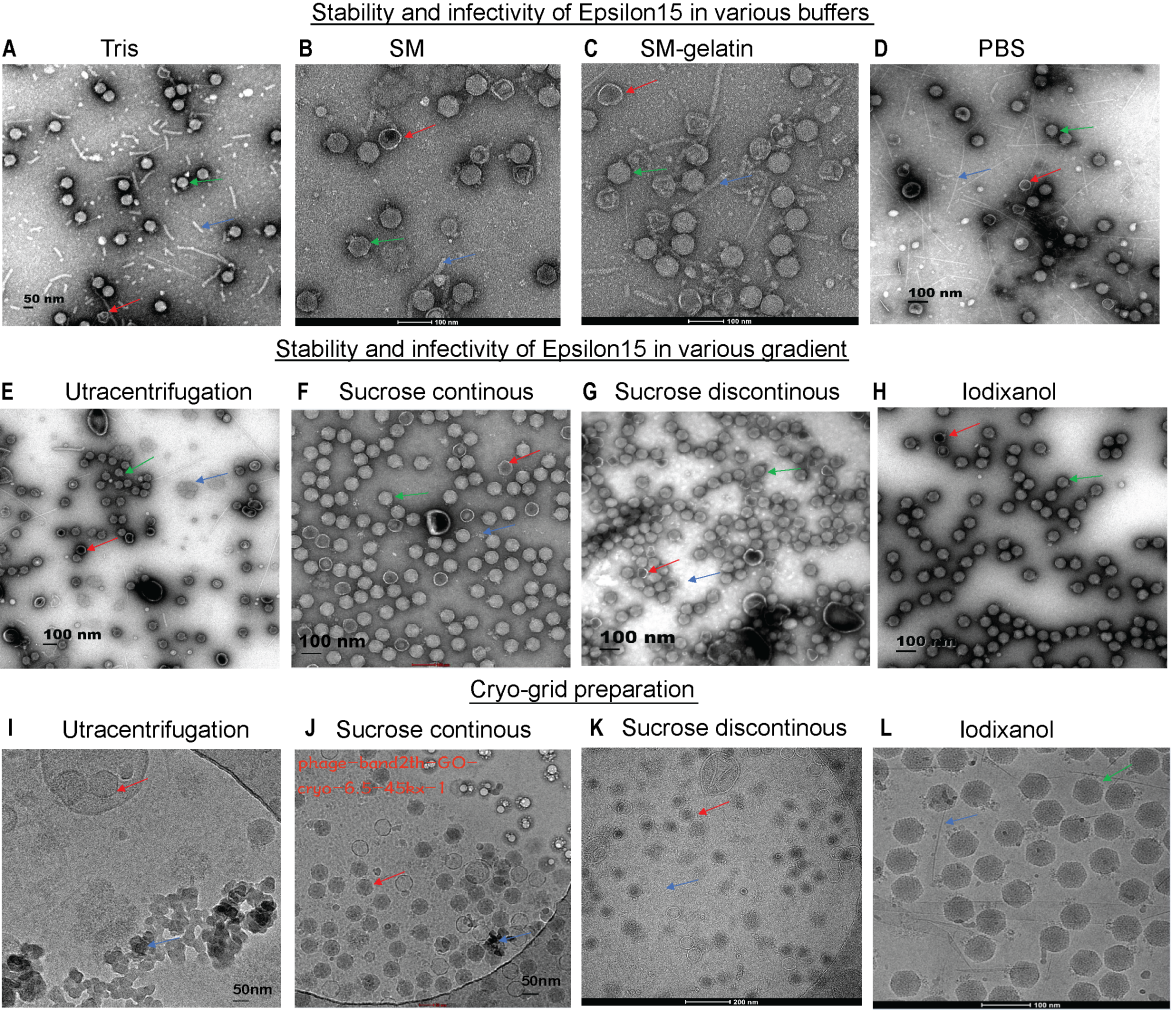
Assessment of ϵ15 virion stability under different purification conditions via EM/Cryo-EM grid preparation. **(A–C)** Negative-staining EM of ϵ15 particles purified using different buffer systems: **(A)** Tris, **(B)** SM buffer, **(C)** SM-gelatine, and **(D)** PBS. **(E–H)** Negative-staining EM of ϵ15 particles following density gradient centrifugation using: **(E)** ultracentrifugation, **(F)** continuous sucrose, **(G)** discontinuous sucrose, **(H)** discontinuous iodixanol gradients **(I–L)** Cryo-EM of ϵ15 virions vitrified on GO-coated grids following purification by: **(I)** ultracentrifugation, **(J)** continuous sucrose, **(K)** discontinuous sucrose, and **(L)** discontinuous iodixanol gradients. Red arrows indicate broken particles; green arrows denote intact virions; blue arrows highlight background debris. Scale bars are indicated 50, 100 or 200 nm in each EM panel.

Optimized purification and preparation protocols preserved ϵ15 virion integrity and infectivity, enabling high-resolution structural and functional analysis. Structural resilience of ϵ15 varied significantly across tested buffers, with SM buffer (**Figure 1B**) providing superior preservation of intact capsid-tail complexes and minimal debris compared to Tris and PBS. Tris buffer resulted in extensive particle breakage, detached tails, and high background debris (**Figure 1A**), while PBS produced heterogeneous populations with partial capsid collapse, moderate tail loss, and aggregation (**Figure 1D**). The addition of gelatin to SM buffer further enhanced structural integrity, yielding monodisperse, intact virions with virtually no fragments (**Figure 1C**). However, despite its visual advantages, SM + gelatin did not improve infectivity, with PFU titers comparable to those of PBS and SM, and it posed challenges for cryo-EM grid preparation due to potential imaging artifacts (**Supplementary Figure 1A**). Therefore, PBS was selected for downstream assays, as it balances between particle stability and infectivity, despite showing slightly reduced morphological preservation.

The choice of purification method profoundly influenced ϵ15 virion integrity and cryo-EM sample quality. Negative-stain EM revealed that ultracentrifugation and discontinuous sucrose cushions caused extensive particle aggregation, fragmentation, and capsid disruption (**Figures 1E, G**). Continuous sucrose gradients modestly improved particle preservation, with a higher number of intact virions, though structural damage remained common (**Figure 1F**). In contrast, iodixanol gradients yielded the highest-quality preparations, with the highest number of virions retaining intact capsid-tail complexes and densely packed genomes (**Figure 1H**). These preparations also exhibited superior infectivity, significantly outperforming other methods (*P* < 0.0001), while no notable differences were observed among sucrose-based or ultracentrifugation preparations (**Supplementary Figure 1B**). The buoyant density of ϵ15 differed across all gradient media, as shown in **Supplementary Figures 1C–E**.

Cryo-EM grid assessments confirmed that iodixanol-purified ϵ15 samples offered optimal quality for high-resolution structural analysis (**Figure 1L**). Vitrification on GO-coated grids enabled uniform particle adsorption and diverse orientations, which are essential for accurate three-dimensional reconstructions. Ultracentrifugation-purified samples exhibited severe aggregation and high background noise (**Figure 1I**), while sucrose gradient preparations showed better dispersion but were compromised by residual debris and capsid damage (**Figures 1J, K**). In contrast, iodixanol-purified particles remained monodisperse, structurally intact, and were imaged against a clean background, facilitating accurate particle picking and efficient downstream processing. These results establish iodixanol purification as the optimal method for preserving ϵ15 integrity, offering the best balance of structural quality and functional suitability for single-particle cryo-EM studies.

### 3.2 Structural elucidation of the ϵ15 and its DNA packaging apparatus

**Figure 2:**
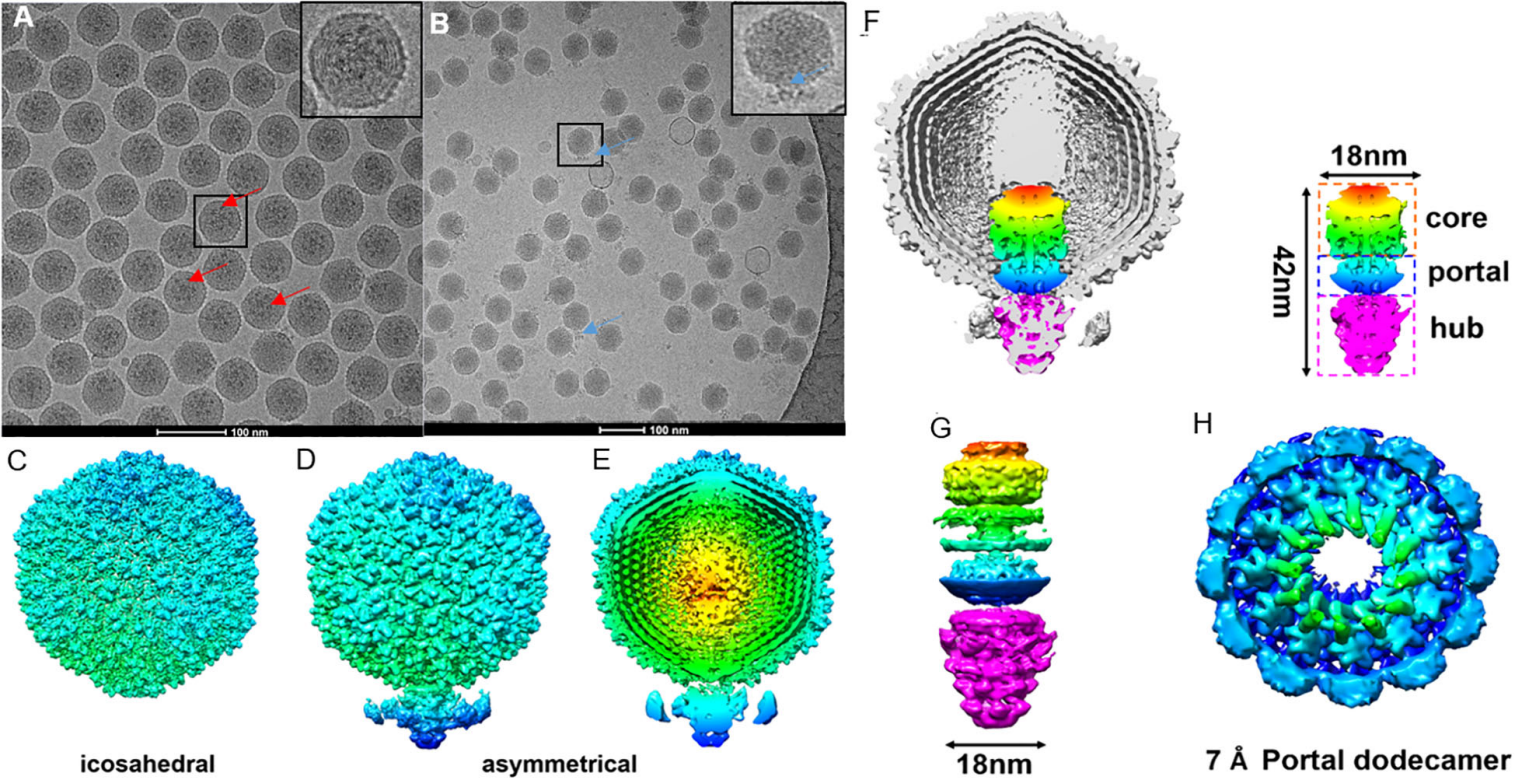
Cryo-EM grid support evaluation and asymmetric reconstruction of the genome packaging machinery in ϵ15. **(A)** Cryo-EM micrographs of ϵ15 particles vitrified on a standard holey carbon grid, exhibiting strongly preferred orientation. **(B)** Cryo-EM micrographs of ϵ15 prepared on a perforated grid coated with a GO, demonstrating improved particle dispersion and orientation. **(C)** Icosahedral 3D reconstruction of the capsid, revealing the symmetric protein shell architecture. **(D)** Asymmetrical reconstruction of the full virion, capturing the tail complex anchored at a unique capsid vertex. **(E)** Cross-sectional view of the asymmetric map, with radial coloring highlighting concentric layers of packaged genomic DNA. **(F)** Cutaway view of the full reconstruction, illustrating the ordered DNA arrangement and the portal-tail complex. The schematic outlines three main components: core, portal, and hub regions (~42 nm total length, ~18 nm core width). **(G)** Side view of the portal-tail complex density map, shown in both intact and exploded views corresponding to the structural elements in panel **(H)** Focused 7 Å reconstruction of the portal region, revealing a dodecameric ring composed of 12 subunits critical for DNA translocation. Red arrow: the tail of ϵ15 aligned with the icosahedral axis.; Blue arrow: the tail of ϵ15 positioned at the side of the icosahedron. Scale bar: 200 nm for panels **(A, B)**.

To uncover the native architecture of ϵ15 and its DNA packaging machinery, high-resolution cryo-EM analysis was performed with a focus on resolving asymmetric and functionally relevant structural features. Two types of cryo-EM grids, either with or without a GO-support film, were used for vitrification. Grids lacking the support film, ϵ15 particles exhibited a strong preferred orientation, limiting structural information during data processing (**Figure 2A**). To address this, an optimized cryo-EM workflow was developed, combining iodixanol gradient purification to enhance particle integrity with GO-coated grids to reduce orientation bias and support-fragile appendages in vitreous ice. This dual strategy yielded high-quality micrographs of intact, well-dispersed ϵ15 virions for single-particle reconstruction (**Figure 2B**), enabling comprehensive structural analysis. An initial icosahedral reconstruction resolved the symmetric capsid at high resolution but, as expected, did not capture the asymmetric unique tail vertex (**Figure 2C**).

Asymmetric single-particle reconstruction was implemented to resolve the complete ϵ15 virion (**Figure 2D**), successfully visualizing the ~42 nm portal-tail complex anchored at a unique capsid vertex, along with distinct internal densities corresponding to the packaged genome. Cross-sectional analysis of the asymmetrical map revealed at least eight concentric DNA layers and arranged radially from the center and approximately 28 longitudinally stacked layers, underscoring the highly ordered organization of the phage genome (**Figure 2E**). The portal-tail machinery was clearly resolved into three modules: a central portal ring, an upper core, and a lower hub, measuring ~18 nm in width and ~42 nm in length (**Figures 2F, G**). Focused refinement of the central portal yielded a dodecameric architecture at ~7 Å resolution (**Figure 2H**), consistent with conserved portal structures in tailed phages. In contrast, the tail hub exhibited a hexameric organization, indicating structural specialization for DNA translocation.

Together, these results bridge longstanding gaps between capsid symmetry and asymmetric functional components. By overcoming sample fragility and orientation bias, the *in-situ* DNA packaging machinery and internal genome organization of ϵ15 were resolved in unprecedented detail. This integrated structural view reinforces ϵ15 as a tractable model for dissecting the molecular choreography of tailed phage infection and genome delivery. ϵ15 is a short-tailed dsDNA bacteriophage that infects *Salmonella* species. During infection, it deploys a series of enzymatic proteins to mediate host attachment, genome translocation, replication, and eventual progeny release. Among these, the endolysin encoded by gene product LysSA05 is implicated in degrading the bacterial cell wall during the final stage of the lytic cycle. Although its exact mechanism remains to be fully defined, LysSA05 may possess unique structural elements that enable it to penetrate the outer membrane of Gram-negative bacteria, a rare but valuable capability among phage-derived enzymes. Characterizing its function will advance our understanding of ϵ15 biology and support its development as an antimicrobial agent.

### 3.3 LysSA05 binding specificity toward NAM and NAG disaccharides

**Figure 3:**
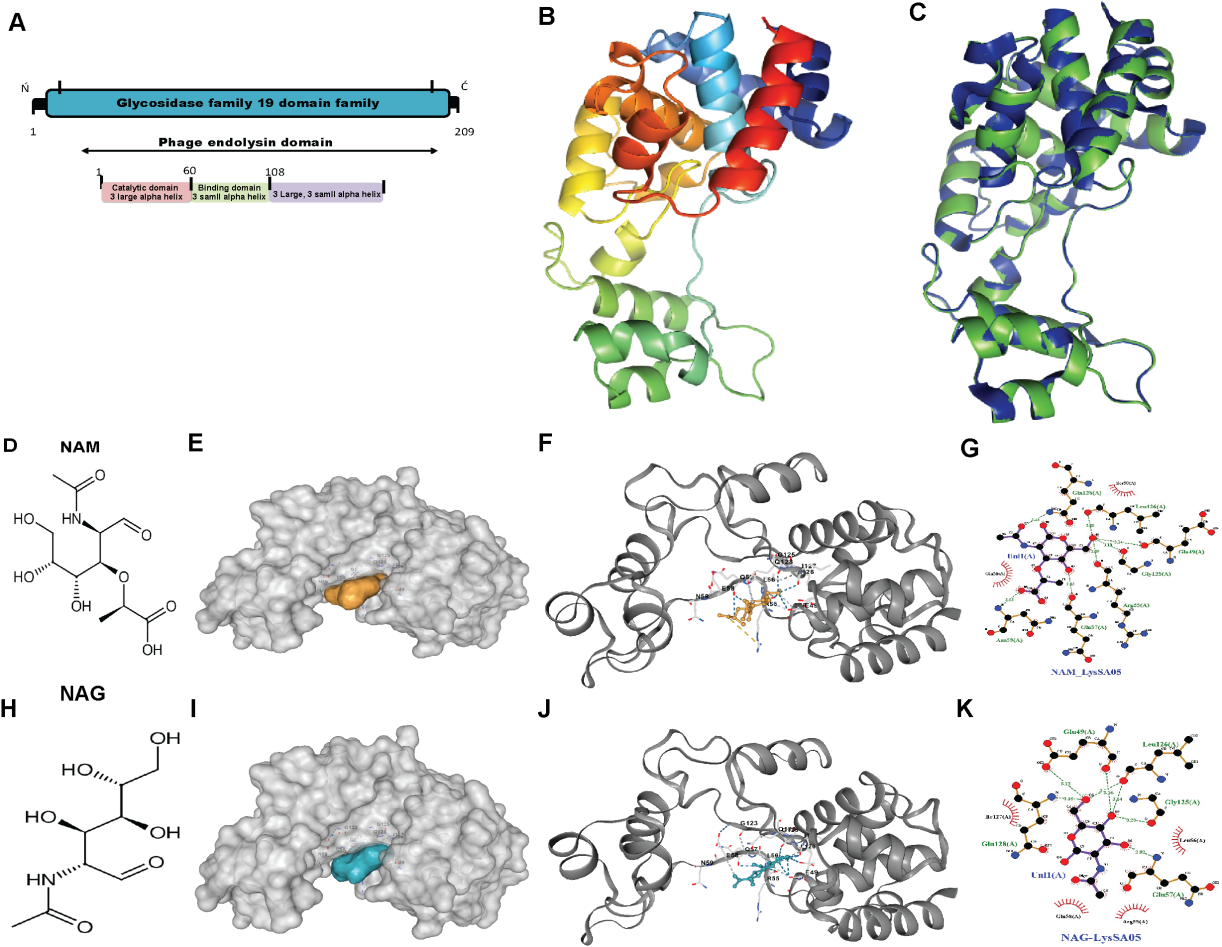
Structural features and molecular docking of LysSA05 endolysin with peptidoglycan ligands NAM and NAG. **(A)** Domain architecture of LysSA05 endolysin, highlighting the glycosidase family 19 (GH19) catalytic domain (pink) and the peptidoglycan-binding domain (green), along with predicted α-helical segments. **(B)** Ribbon diagram of the predicted 3D structure of LysSA05, showing domain organization and two prominent groove loops. **(C)** Superimposition of the predicted LysSA05 structure with a homologous template (PDB: 4OK7), shown in blue. **(D)** 2D chemical structure of NAM. **(E)** Surface representation of LysSA05 with NAM-binding cavity highlighting in yellow. **(F)** NAM docked into catalytic groove of LysSA05 (cartoon view); key interactions are shown as dashed lines. **(G)** Zoomed-in view of the NAM binding pocket, showing hydrogen bonds and hydrophobic contacts stabilizing the interaction. **(H)** 2D chemical structure of NAG. **(I)** Surface representation of LysSA05 with the NAG-binding pocket highlighted in blue. **(J)** NAG docked into LysSA05 cavity, visualizing ligand–protein interactions. **(K)** Close-up of the NAG binding site; showing hydrogen bonds are represented by green bashed lines, while red spokes denote hydrophobic interaction, with bond distance shown in angstrom Å.

The *Salmonella* phage ϵ15 genome encodes a putative endolysin gene, *gp05* (GenBank ID: AAO06088.1), hereafter referred to as LysSA05. This gene encodes a 209-amino-acid protein with an estimated molecular weight of ~25 kDa and a theoretical pI of 9.54. ProtParam analysis yielded an instability index of 21.17 and a GRAVY score of −0.456, suggesting that LysSA05 is a stable, moderately hydrophilic protein. These properties are comparable to those of LysSE24, a dual-acting endolysin reported to be active against Gram-negative bacteria (Ding et al., 2020). BLASTP analysis revealed 94.23% sequence identity to the endolysin of *Salmonella* phage SPN1S (**Supplementary Figure 2A**). Conserved domain searches and phylogenetic analysis identified a GH19 catalytic domain spanning residues 12-208 (**Supplementary Figure 2C**). Secondary structure prediction using PSIPRED and PHD indicated that LysSA05 is predominantly α-helical (57.89%) with notable coil regions (38.76%), including a predicted transmembrane helix between residues 39-54 (**Supplementary Figure 2B**). Functional annotation revealed a modular architecture comprising a peptidoglycan binding domain (PBD) and a peptidoglycan cleaving domain (PCD). HHpred analysis further classified the protein within the CAZy GH19 family (E-value = 12, probability = 83.6%), supporting its potential to hydrolyze β-1,4 glycosidic bonds between NAM and NAG within bacterial cell walls (**Figure 3A**). Tertiary structure modeling using Phyre2 selected the endolysin from *S. Typhimurium* phage SPN1S (PDB ID: C4OK7A) as the optimal structural template (**Figure 3B**). The predicted model showed a root mean square deviation (RMSD) of 0.085 relative to the template, indicating strong structural similarity (**Figure 3C**). Model validation by ERRAT yielded a quality score above 95%, while Ramachandran plot analysis showed 91% of residues in favored regions, collectively, supporting the reliability and accuracy of the predicted LysSA05 structure (**Supplementary Figure 2D**). To investigate the molecular basis of PG recognition by LysSA05 endolysin, cavity-guided blind docking was performed using CB-Dock2 with two critical PG monomers: NAM and NAG. Docking of NAM produced the top-ranked pose in the largest cavity (Vina score: -5.3 kcal/mol) (**Figures 3D–G**), where NAM was deeply embedded into a hydrophilic pocket lined by residues E49, R55, S50, and Q128. Multiple hydrogen bonds and hydrophobic contacts with I126 and L56 stabilized the ligand orientation within the groove. Similarly, NAG docking identified a favorable cavity (Vina score: -5.3 kcal/mol), with five hydrogen bonds formed with E49, S50, L55, L56, Q57, and E58. Additional hydrophobic interactions with N59, G123, G125, Q128, L126, and I127 contributed to ligand stabilization within a polar-hydrophobic binding pocket (**Figures 3H–K**). The observed binding poses and interaction geometries suggest that both NAM and NAG occupy functionally relevant substrate-binding pockets in LysSA05, mimicking native PG recognition. These insights provide a structural rationale for future mutational or inhibitor design. As with others GH19 family glycoside hydrolases, the active site is shaped to accommodate NAM-NAG disaccharides, enabling precise positioning of the scissile bond β-1,4 glycosidic for hydrolysis. The substrate is stabilized through a conserved network of hydrogen bonding and hydrophobic contacts within the catalytic groove, underscoring the structural and functional potential of LysSA05 as a PG-degrading enzyme.

### 3.4 Antimicrobial propensity and LysSA05 derived AMP peptides

**Figure 4:**
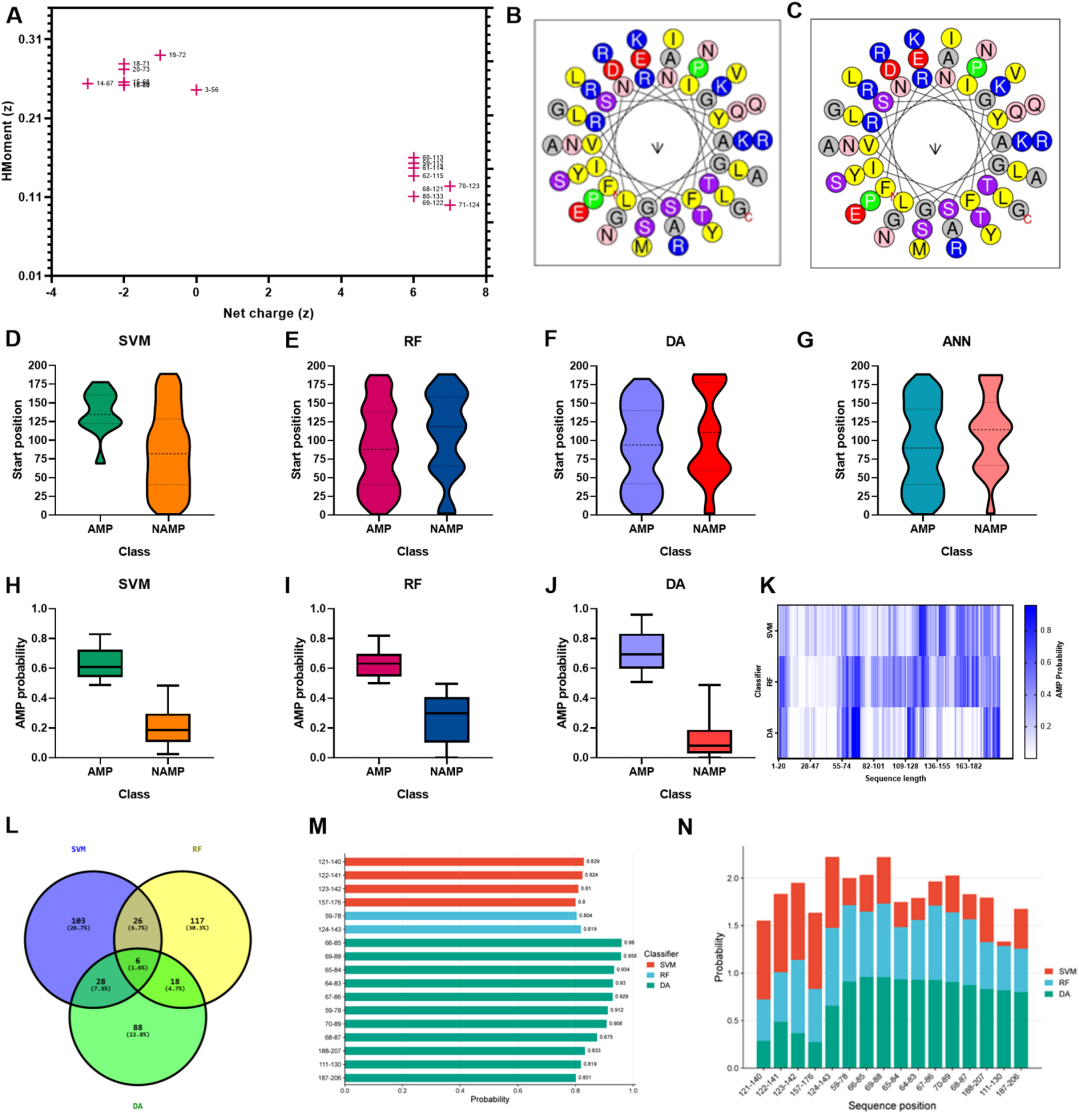
AMP prediction from LysSA05 using machine learning and physicochemical profiling. **(A)** Preliminary-HeliQuest analysis showing net charge (z) and hydrophobic moment (µH) of predicted helical segments. **(B–C)** Helical wheel projections of top two amphipathic helices, illustrating residue polarity and spatial distribution. **(D–G)** Distribution of AMP and NAMP prediction across helix position based on four machine learning classifiers: SVM, RF, DA, and ANN. **(H–J)** AMP probabilities scores for predicted segments; AMP-classified helices consistently show significantly higher probability across all the models. **(K)** Heatmap representing AMP probabilities scores across the LysSA05 sequence, highlighting regions with high AMP potential. **(L)** Venn diagram illustrating the overlap of AMP predictions among SVM, RF, and DA classifiers. **(M)** Top- ranked AMP helices predicted by SVM, RF, and DA with probability scores ≥ 0.8. **(N)** Final selection of high-confidence AMP helices in LysSA05 identified across the three classifiers (SVM, RF, DA), each with AMP probability ≥ 0.8.

To explore alternative therapeutics against MDR pathogens, the endolysin LysSA05 from phage ϵ15 was screened for membrane-active, AMP-like regions. Initial physicochemical analysis using HeliQuest on a 54-residue segment identified 155 helix positions ranked by amphipathic potential (**Supplementary Table 3**). Of these, 17 distinct amphipathic helices displayed pronounced AMP-like characteristics, marked by elevated hydrophobic moment (µH) and net positive charge (z), indicative of strong membrane-disruptive potential (**Figure 4A**). Notably, helices at positions 59 and 60 exhibited optimal µH values (≥ 0.15) and high net charge (+6), suggesting robust amphipathic properties (**Figures 4B, C**) and a potential role as functional AMP domains capable of destabilizing bacterial membranes. Further screening using machine learning classification of 20-residue segments across 189 peptide positions revealed varying counts of AMP-positive predictions: RF identified the most (46), followed by DA (35), ANN (34), and SVM (28). Positional mapping of these predictions revealed distinct model-specific distribution patterns. Notably, SVM exhibited a strong positional bias towards the N-terminal to mid-sequence, while other models detected AMP-like regions more broadly across the protein (**Figures 4D–G**). To further validate the predicted antimicrobial regions within LysSA05, the probability scores assigned by each classifier to AMP and non-AMP (NAMP) segments were analyzed. Peptides classified as AMPs consistently exhibited significantly higher probability scores than NAMPs across all models (SVM, RF, and DA), indicating high classifier confidence (**Figures 4H–J**). Among these, the DA model demonstrated the strongest discriminatory power, with AMP segments clustering near probability values > 0.9, while NAMPs remained below 0.4. A heatmap analysis revealed the spatial probability distribution of AMP probability scores across the LysSA05 sequence, highlighting recurrent high-probability regions between residues 60–90 and 120-140 (**Figure 4K**). Notably, the top-ranked AMP segments identified by each classifier included helix 121-140 (SVM, 0.829), helix 124-143 (RF, 0.819), helix 66-85 (DA, 0.96). Although these high-scoring helices varied slightly in position across classifiers, they converged in identifying two AMP-enriched regions. Despite the strong performance of individual classifier, overlap among the top predictions was limited: only six sequences (1.6%) were shared across all three models (**Figure 4L**). Applying a probability threshold of ≥ 0.8, 4 helices from SVM, 2 from RF, and 11 from DA were identified (**Figure 4M**). Interestingly, none of the high-confidence predictions overlapped at the same helix position across models, suggesting that each classifier captured distinct AMP-relevant features. Among these top candidates, the helices 59-78, 65-84, 67-86, and 188-207 were consistently assigned high scores by multiple models, representing promising antimicrobial targets (**Figure 4N**). Collectively, these findings indicate that LysSA05 harbors several AMP-like regions with distinct location-specific enrichment patterns, which may contribute to its membrane-disruptive activity and broad-spectrum antibacterial potential.

### 3.5 Amphipathic and structural features of top-ranked AMP-like peptides from LysSA05 endolysin

**Figure 5:**
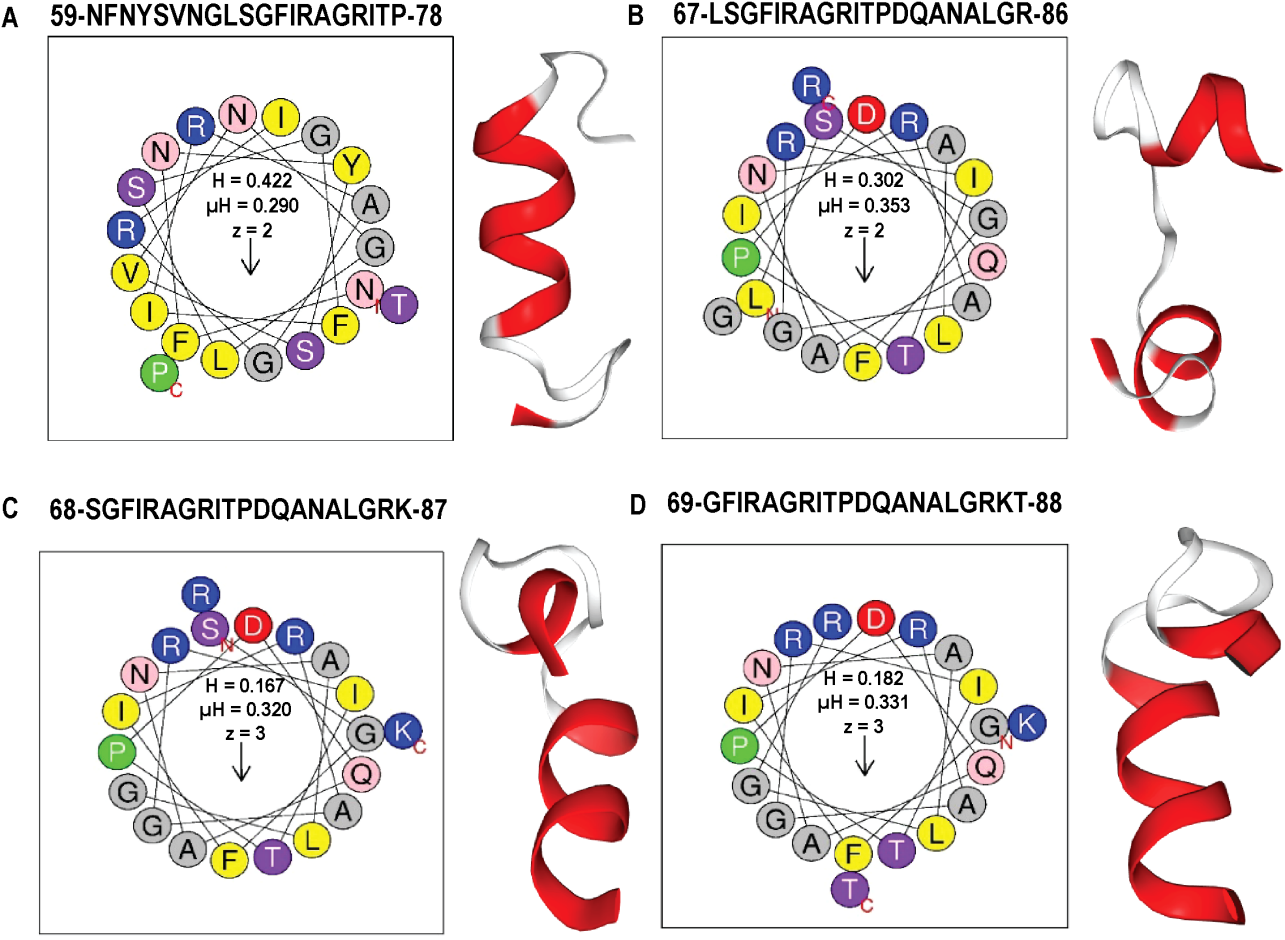
Amphipathic properties and α-helical structures of top AMP-like peptides derived from LysSA05 endolysin. **(A–D)** Helical wheel projections and physicochemical parameters for four top-ranking peptides **(A)** 59–78, **(B)** 67–86, **(C)** 68–87, and **(D)** 69–88 generated using HeliQuest. Each projection illustrates the distribution of residues around the helical axis, revealing distinct amphipathic separation: polar/charged residues (blue/purple) cluster on the solvent-exposed face, while hydrophobic residues (yellow/grey) align on the membrane-interacting face. Accompanying boxes summarize key parameters: hydrophobicity **(H)**, hydrophobic moment (µH), and net charge (z). The 3D ribbon diagram (left panels) shows predicted α-helical confirmation of each peptide, highlighting their structural potential for membrane insertion and antimicrobial activity.

By integrating ML-based classification (**Supplementary Tables 4–9**, **Table 1**) with physicochemical screening using HeliQuest (**Supplementary Table 10**), four outstanding candidate peptides helix 59 (z = +2, µH = 0.29), helix 67 (z = +2, µH = 0.353), helix 68 (z = +3, µH = 0.32), and helix 69 (z = +3, µH = 0.331) were identified that exhibited both high AMP probabilities and strong amphipathic character. These candidates were selected from comprehensive segmentation of 20-residue window across 189 helical positions (**Supplementary Table 11**). To assess their membrane-targeting potential, helical wheel projections and 3D ribbon modeling were performed. The peptides corresponding to positions 59-78, 67-86, 68-87, and 69–88 displayed well-define amphipathic α-helical structures, characterized by a clear segregation of hydrophobic residues (F, L, I, A, G, V) on one face and positively charged residues (R, K) on the opposite hydrophilic face, a hallmark of membrane active AMPs. Helical wheel diagrams confirmed that these peptides exhibit ideal biophysical properties for membrane disruption, with hydrophobic moments ranging from 0.29 to 0.353 and net charges between +2 and +3. 3D modeling of peptide 59–78 further supported a stable α-helical conformation, consistent with its predicted role in membrane interaction (**Figures 5A–D**). These structural features parallel those found in previously characterized broad-spectrum phage endolysins known to possess outer membrane-permeabilizing activity. Collectively, these findings highlight the central region of LysSA05 spanning residues 59-112, as a key AMP-like domain with dual functional potential: enzymatic degradation of PG via NAM-NAG interactions, and direct disruption of the Gram-negative outer membrane through embedded amphipathic helices. This dual-action model underscores LysSA05’s promise as a broad-spectrum enzybiotic candidate.

**Table 1:** Top-ranked helical segments in LysSA05 with AMP probability scores ≥ 0.8 across as predicted by three machine learning classifiers: (SVM, RF, DA, and ANN).

Position	Sequence segment	SVM	RF	DA	ANN
121-140	RGRGLIQITGLNNYRDCGNG	0.829	0.436	0.287	AMP
122-141	GRGLIQITGLNNYRDCGNGL	0.824	0.522	0.488	AMP
123-142	RGLIQITGLNNYRDCGNGLK	0.81	0.772	0.368	AMP
157-176	YAARSAAWFFATKGCMKYTG	0.8	0.561	0.274	AMP
124-143	GLIQITGLNNYRDCGNGLKV	0.746	0.819	0.658	AMP
59-78	NFNYSVNGLSGFIRAGRITP	0.285	0.804	0.912	AMP
66-85	GLSGFIRAGRITPDQANALG	0.385	0.689	0.96	AMP
69-88	GFIRAGRITPDQANALGRKT	0.488	0.775	0.958	AMP
65-84	NGLSGFIRAGRITPDQANAL	0.262	0.551	0.934	AMP
64-83	VNGLSGFIRAGRITPDQANA	0.231	0.63	0.93	NAMP
67-86	LSGFIRAGRITPDQANALGR	0.253	0.783	0.929	AMP
70-89	FIRAGRITPDQANALGRKTY	0.388	0.732	0.908	AMP
68-87	SGFIRAGRITPDQANALGRK	0.263	0.692	0.875	AMP
188-207	GQNGIDDRRARYITASKVLA	0.468	0.493	0.833	AMP
111-130	GNGPGDGWNYRGRGLIQITG	0.045	0.467	0.819	AMP
187-206	GGQNGIDDRRARYITASKVL	0.418	0.457	0.801	AMP

### 3.6 Expression optimization and purification of recombinant endolysin LysSA05

**Figure 6:**
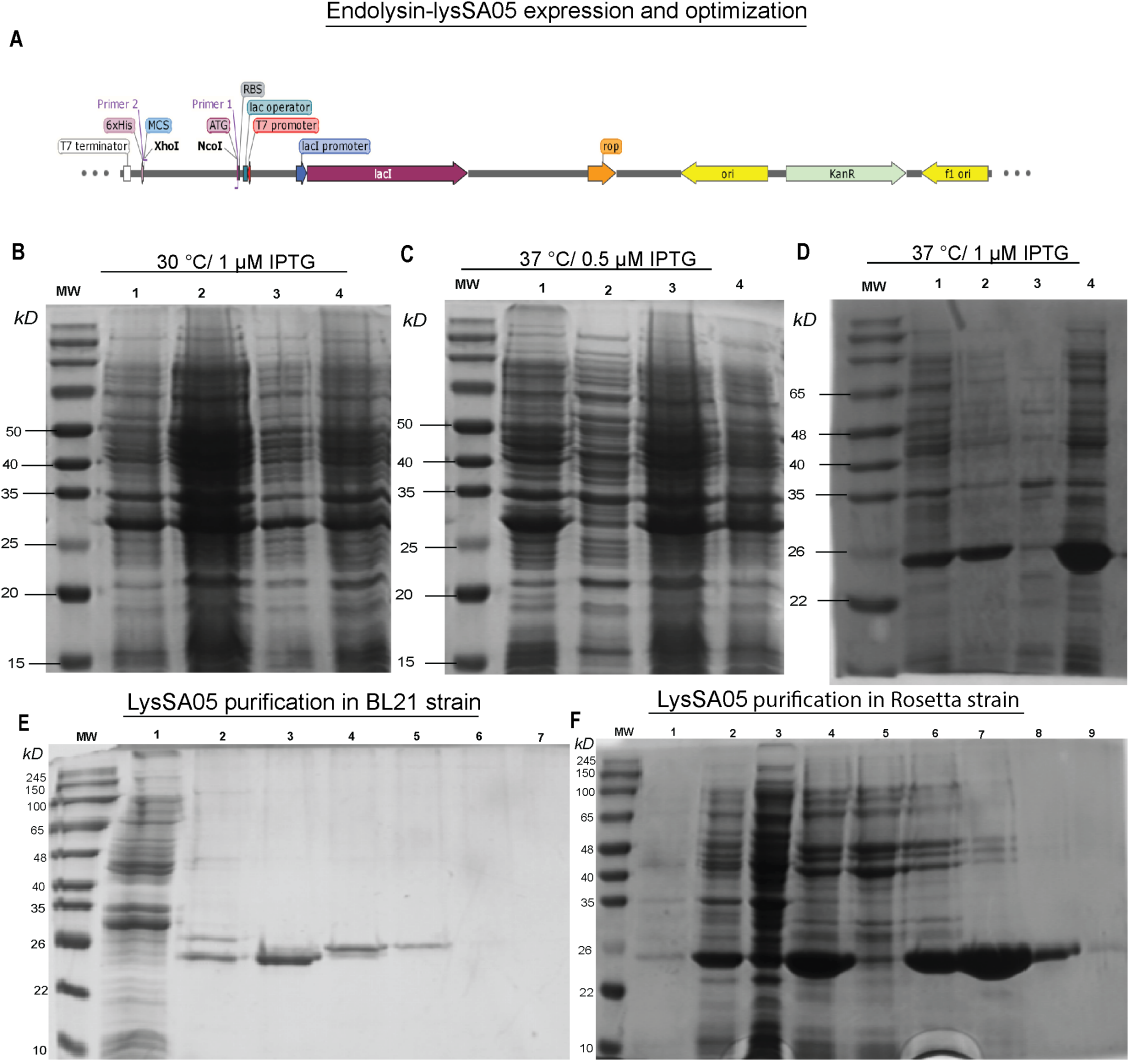
Expression optimization and purification of recombinant LysSA05. **(A)** Schematic diagram illustrating the cloning of *gp05* into the pET-28a(+) vector using *NcoI* and *XhoI* restriction sites. **(B–D)** SDS-PAGE analysis of expression optimization in *E. coli* Rosetta under varying conditions: **(B)** 30°C with 1 mM IPTG; **(C)** 37°C with 0.5 mM IPTG; **(D)** 37°C with 1 mM IPTG. A distinct ~25 kDa band corresponding to His_6_-tagged LysSA05 is observed in lane 4 (Rosetta, 37°C, 1 mM IPTG), indicating optimal expression. **(E)** Purification of LysSA05 from *E*. *coli* BL21 showing low yield after Ni-NTA affinity chromatography across elution fractions 3-6, compared to control (lanes 1, 2). **(F)** Purification from Rosetta strain showing high expression and high >90% purity of the ~25 kDa protein across elution fractions 5-9. Lane 1: non-induced control; Lane 2: supernatant after centrifugation; Lane 3: soluble fraction post-sonication; Lane 4: total cell lysate post-sonication.

To facilitate downstream functional validation of LysSA05 as a membrane-disruptive, AMP-like endolysin, the *gp05* gene encoding LysSA05 was successfully cloned and sequence-verified (**Figure 6A** shows the schematic representation; **Supplementary Figures 3A-C** confirms successful cloning). Expression analysis revealed a ~25 kDa band consistent with the predicted molecular weight of LysSA05, with markedly stronger expression observed in *E. coli* Rosetta (DE3) induced with 1 mM IPTG at 37°C (**Figures 6B–D**), compared to BL21 (DE3) (**Supplementary Figure 3D, E**). The protein was localized exclusively in the insoluble fraction, indicating the formation of inclusion bodies under all tested conditions. Subsequent purification yielded highly enriched LysSA05 protein from Rosetta (**Figure 6F**), in contrast to lower yield from BL21 (**Figure 6E**). Final protein concentrations reached ~11 mg in Rosetta (DE3), significantly exceeding the ~2.5 mg obtained from BL21. The refolded protein with > 90% purity was stable and of sufficient quality for downstream biochemical assays. These findings confirm Rosetta as a more efficient expression host and provide a robust production system for high-purity LysSA05. This enables comprehensive investigation of its dual functional potential: enzymatic cleavage of PG and membrane-disruptive activity predicted through machine learning and structural modeling approaches.

### 3.7 Characterization of LysSA05 activity

**Figure 7:**
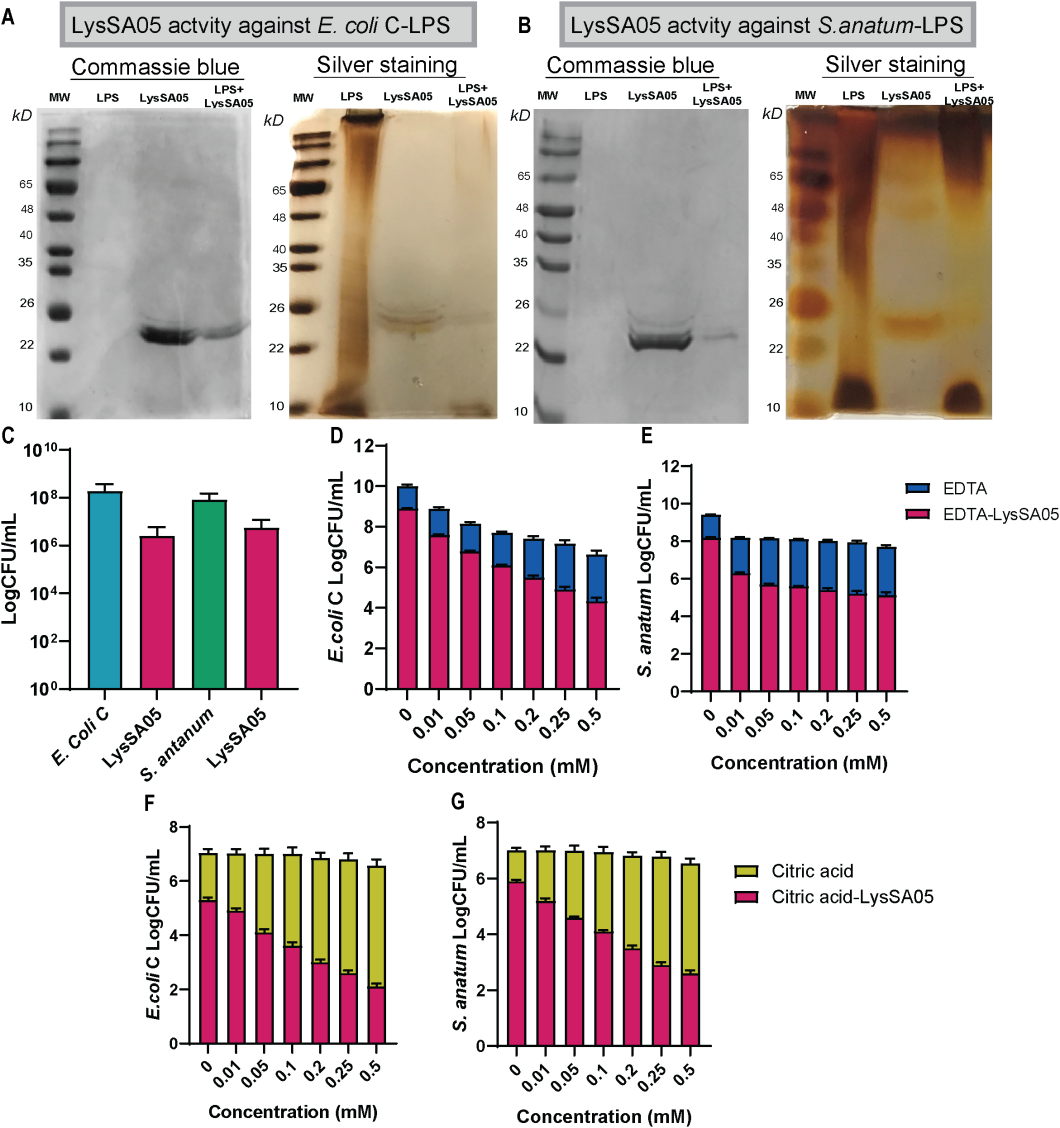
Characterization of LysSA05 LPS-degrading activity and enhancement of antibacterial activity by OMPs. **(A)** SDS-PAGE and silver staining demonstrating LPS degradation in *E*. *coli* C following treatment with LysSA05. **(B)** SDS-PAGE and silver staining showing LPS degradation in *S. anatum* treated with LysSA05. **(C)** CFU reduction assay indicating the direct antibacterial effect of LysSA05 against *E*. *coli* C and *S. anatum*, with log reductions of approximately 2.0 and 1.3, respectively, after 5 h of incubation. **(D, E)** Synergistic effect of various EDTA concentrations (mM) in combination with LysSA05 against *E. coli* C and *S. anatum*. **(F, G)** Synergistic effect of different citric acid concentrations (mM) in combination with LysSA05 against *E*. *coli* C and *S. anatum*.

The lytic activity of full-length recombinant LysSA05 was evaluated against Gram-negative strains *E. coli* C and *S. anatum*. SDS-PAGE followed by silver staining of extracted LPS matrices revealed pronounced degradation upon LysSA05 treatment, with a marked reduction in LPS band intensity in *E. coli* C compared to untreated controls (**Figures 7A, B**). These findings indicate that LysSA05 can directly disrupt LPS, a key structural component of Gram-negative outer membrane. Complementary CFU reduction assays further demonstrated LysSA05’s intrinsic lytic activity, showing a ~2.0 log_10_ reduction in *E. coli* C and ~1.3 log_10_ in *S. anatum* after 5 h (**Figure 7C**). Notably, co-treatment with OMPs (EDTA and citric acid) significantly enhanced LysSA05-mediated lysis. In combination with EDTA, bacterial killing increased to ~4.5 log_10_ in *E. coli* C and ~3.4 log_10_ in *S. anatum* (**Figures 7D, F**), while citric acid produced comparable enhancement (**Figures 7E, G**). These results strongly support the presence of membrane-active elements in LysSA05, likely associated with its amphipathic α-helical regions, and highlight its capacity to overcome the intrinsic barrier imposed by Gram-negative outer membranes. Collectively, these findings support the hypothesis that full-length LysSA05 functions as a dual-acting enzybiotic, exhibiting both membrane-permeabilizing activity, experimentally confirmed through LPS degradation assays and CFU reduction assay, and PG-cleaving potential, inferred from GH19 domain modeling and ligand docking with NAM and NAG. This dual functionality underscores its promise as a therapeutic candidate against MDR Gram-negative pathogens.

### 3.8 Antibacterial activity of LysSA05 against MDR clinical isolates

**Figure 8:**
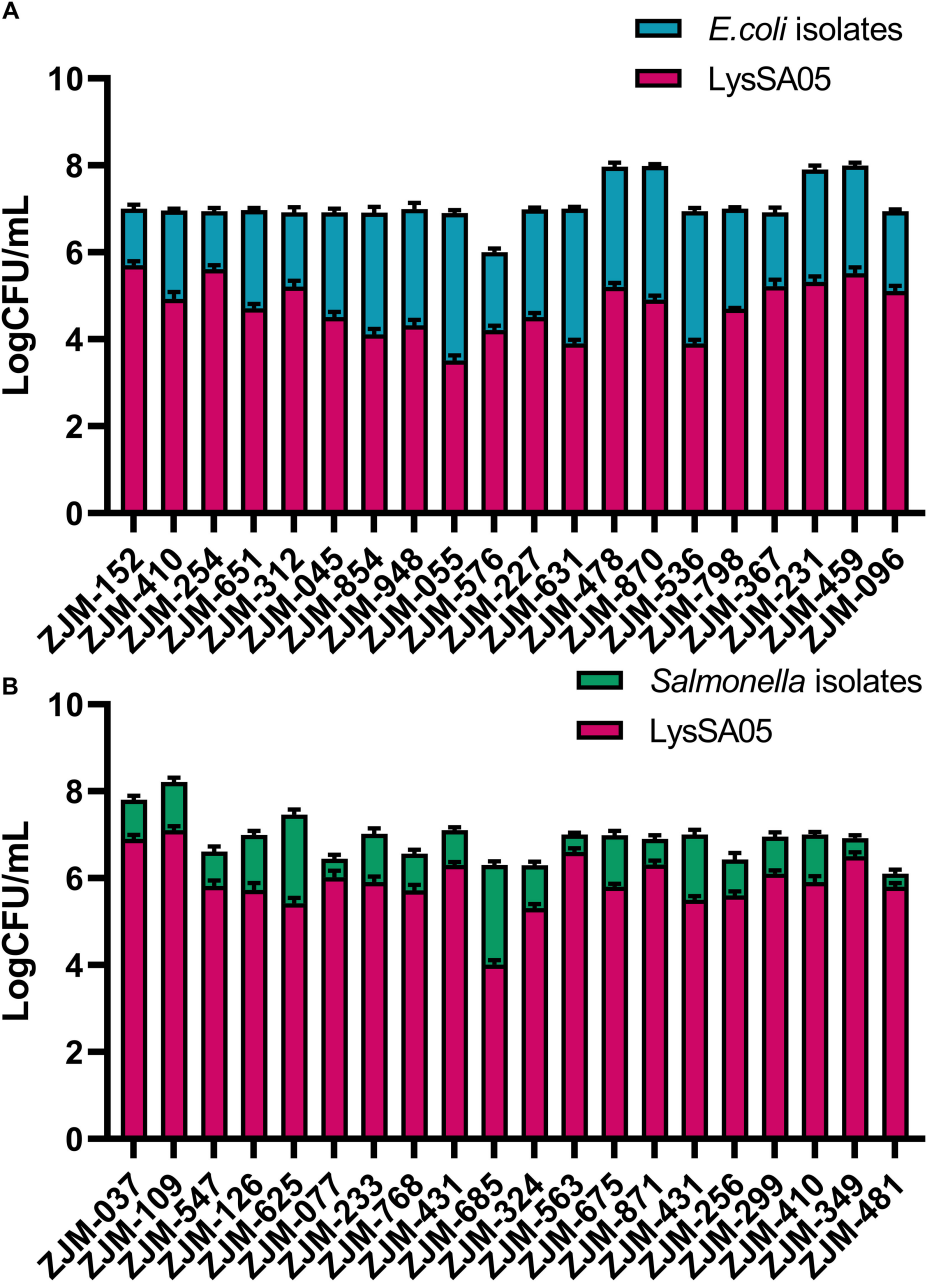
Antimicrobial activity of LysSA05 against clinical isolates of *E. coli* and *Salmonella*. **(A)**. Bactericidal effect of LysSA05 (100 μg/ml) against logarithmic-phase *E. coli* clinical isolates following 1 h incubation at 37°C. **(B)** Bactericidal effect of LysSA05 (100 μg/ml) against logarithmic-phase *Salmonella* clinical isolates under the same conditions.

To evaluate the broader antimicrobial potential of LysSA05, we tested the activity of full-length recombinant protein, containing predicted AMP-like regions, against a panel of MDR clinical isolates of *E. coli* and *Salmonella* spp., with diverse resistance profiles (**Supplementary Table 2**). The CFU-based plating assays were conducted to quantify bacterial viability following treatment with LysSA05. LysSA05 exhibited broad-spectrum lytic activity against all tested *E. coli* clinical isolates, achieving reductions in viable cell counts ranging from 1.0 to 4.5 log units (**Figure 8A**). Similarly, all *Salmonella* isolates tested were susceptible to LysSA05, though to a lesser extent, showing reductions between 0.5 and 3.0 log units (**Figure 8B**). The observed variability in susceptibility likely reflects differences in outer membrane composition or LPS structure among strains, which can affect the accessibility and binding efficiency of membrane-active peptides. These findings highlight the broad-acting antibacterial potential of LysSA05, particularly its efficacy against MDR *E. coli*, and suggest that its therapeutic utility may extend beyond the classical Gram-positive targets typically associated with endolysins.

## 4 Discussion

Bacteriophages and their lytic enzymes have emerged as promising alternative antibacterial strategies to combat the escalating threat of antibiotic-resistant foodborne pathogens (Shah et al., 2023). However, their clinical application remains limited due to challenges such as the presence of endotoxins, residual cellular debris, and incomplete understanding of how phage structural integrity relates to infectivity and enzymatic function. Phages rely on precisely coordinated assembly of structural components such as capsid, tail fibers, DNA packaging machinery, alongside enzymatic machinery including holins, endolysins, and depolymerases to successfully infect and lyse bacterial hosts (Kim et al., 2025a; Guliy and Evstigneeva, 2025). In this study, *Salmonella* phage ϵ15 was employed as a model system to refine purification protocols and achieve high-resolution structural visualization. An iodixanol-based density gradient in PBS was optimized to improve phage purification while preserving structural integrity (Hirst and Hutchinson, 2025). Additionally, an enhanced cryo-EM grid preparation method using GO-support film (Fan and Sun, 2022; Chio et al., 2024) enables the capture of intact, highly purified ϵ15 particles with preserved infectivity and native morphology. Unlike traditional CsCl ultracentrifugation, which often compromises phage bioactivity and can lead to partial genome ejection, the iodixanol-based purification approach yielded virions that retained full genomic DNA and correctly assembled tail fibers. Importantly, the purified, intact, and infectious ϵ15 virions were validated through plaque assay, in contrast to many prior structural studies that did not confirm post-purification infectivity (Jiang et al., 2006; Suleman et al., 2022). This optimized method not only enabled near-native, high-resolution visualization of ϵ15 capsid and DNA packaging machinery but also resulted in endotoxin-free phage preparations with potential for therapeutic application. In parallel, the dual-acting endolysin LysSA05 was functionally characterized through integrative structural modelling and enzymatic assays to elucidate how phage-derived enzymes impact bacterial pathogens. Together, these analyses provide new insights into phage-host interactions at both the structural and functional levels, supporting the advancement of phage-based therapeutics and enzybiotics targeting MDR Gram-negative pathogens.

### 4.1 Cryo-EM structural analysis of ϵ15

Cryo-EM analysis of ϵ15 in this study achieved substantially higher and more complete resolution than previous approaches by preserving fully intact virions. By avoiding traditional CsCl-based purification, which often compromises structural integrity, we were able to visualize the near-native icosahedral state of ϵ15 and reconstruct a higher-resolution density map that revealed new insights into its genome packaging architecture. In contrast, earlier reconstructions frequently suffered from partial DNA loss or excluded the asymmetric tail structure. For example, Jiang previously achieved only ~20 Å resolution for the entire ϵ15 virion (Jiang et al., 2006), and later improved to ~4–5 Å resolution through icosahedral averaging, which excluded the tail (Jiang et al., 2008). In this study, asymmetric reconstruction resolved the internal layers of packaged DNA, illustrating the tightly organized nature of the encapsidated genome. Detailed density analysis of the DNA translocation machinery revealed a complex comprising the portal, core, and hub regions. The portal region reached an estimated resolution of ~7 Å, enabling visualization of architectural details previously inaccessible in ϵ15. Notably, multiple concentric layers of DNA were clearly resolved inside the capsid, reflecting the extreme ~3,000-fold compaction required to accommodate the ~40 kb genome. This exceptionally high packing density underscores the immense internal pressure phages must withstand and offers insights into how that stored energy may be harnessed during genome ejection. These findings provide a more complete understanding of ϵ15 structural biology and highlight the value of improved purification and cryo-EM workflows in resolving phage architecture at near-native conditions.

The structure of bacteriophage ϵ15 also elucidates several key mechanistic features of infection and genome delivery. Direct visualization of the small gp10 subunits linking adjacent capsomers on the inner surface of the capsid supports the “molecular staple” hypothesis, wherein these dimers stabilize the capsid lattice under high internal pressure (Baker et al., 2013). Additionally, the portal complex located at a unique capsid vertex was conclusively shown to form a dodecameric (12-fold) ring that interfaces with a hexameric tail hub, an asymmetric interaction consistent with prior biochemical evidence (Guichard et al., 2013). This asymmetry forms a natural gating mechanism for DNA release. The portal-tail apparatus spans approximately 42 nm and functions as a conduit that holds the encapsidated genome under tension, supporting the previously proposed “primed” ejection state model (Jiang et al., 2006). Structural data from ϵ15 imply a spring-loaded mechanism, in which tail fiber engagement with the host cell surface triggers the opening of the portal-hub gate, leading to rapid DNA expulsion. This model aligns with *in situ* observations from cryo-electron tomography (Chang et al., 2010) and mirrors genome release strategies described in other double-stranded DNA viruses, such as herpesviruses. The precise geometry and dimensions of the ϵ15 DNA packaging and ejection apparatus offer quantitative parameters for modeling phage genome confinement and release dynamics. These findings not only clarify fundamental aspects of phage infection but also provide a framework for bioengineering applications involving DNA delivery systems.

These insights not only deepen our understanding of the ϵ15 life cycle but also carry important practical implications. The ability to visualize the complete DNA packaging and injection machinery at high resolution provides a structural blueprint for elucidating phage-host interaction mechanisms and enables structure-guided phage engineering. For example, detailed knowledge of the portal and tail structures opens avenues to modify phages for altered host specificity, enhancing their potential for therapeutic applications. Achieving even higher resolution (2–3 Å) in key regions could uncover fine structural details that govern phage infectivity, timing of lysis, and interactions with bacterial receptors. Moreover, the use of an endotoxin-free GO-supported cryo-EM workflow can be extended to other therapeutic phages, enabling the capture of transient intermediate states during infection, states that have previously been difficult to visualize. Future studies employing the iodixanol-based purification approach may allow real-time visualization of dynamic events such as tail tube extension during DNA injection, thereby bridging the current gap between static structural snapshots and the dynamic nature of phage infection. Such advances will not only enhance our mechanistic understanding of phage biology but also inform the rational design of next-generation phage therapeutics with improved efficacy and safety.

### 4.2 LysSA05 endolysin activity against Gram-negative bacteria

The endolysin LysSA05 from ϵ15 highlights the potential of phage-derived enzymes as antimicrobial agents beyond their conventional role in the phage infection cycle. Typically, phage endolysins act intracellularly at the final stage of the replication cycle, cleaving the PG layer from within to facilitate the release of progeny virions (Guliy and Evstigneeva, 2025). Historically, such enzymes have been most effective against Gram-positive pathogens, whose exposed PG layers are readily accessible. In contrast, the outer membrane of Gram-negative bacteria serves as a formidable barrier, shielding the PG layer and preventing the activity of externally applied endolysins (Zampara et al., 2020). Consistent with this, the majority of endolysins derived from Gram-negative phages exhibit little to no activity unless the outer membrane is first permeabilized by OMPs (Park et al., 2014). To overcome this limitation, bioengineered chimeric endolysins, such as Artilysins and Innolysins, have been developed. These constructs combine enzymatic PG-degrading domains with OMPs, and have demonstrated broad-spectrum activity against Gram-negative pathogens (Yang et al., 2015; Thandar et al., 2016; Murray et al., 2021).

LysSA05 is particularly significant in this context because it naturally bypasses the outer membrane barrier of Gram-negative bacteria, a key limitation for most endolysins. Docking and domain prediction analyses reveal that LysSA05 consists of a PG-cleaving glycosidase domain and a C-terminal amphipathic peptide region. Sequence homology indicates that its catalytic domain belongs to the GH19, consistent with endolysins such as LysSPN1S, PlyG, and LysCP28, which cleave PG glycan chains (Lu et al., 2023). Building upon recent advances in AMP-ESKtides databases curated from phage genomes (Wu et al., 2024), the C-terminal region of LysSA05 was identified as highly cationic and hydrophobic. This same 20-amino acid segment was predicted by multiple machine learning tools to be membrane-active and shares strong similarity with motifs found in broad-spectrum endolysins such as Abtn-4 and SPN1S, known for their activity against both Gram-positive and Gram-negative pathogens. Computational modeling suggests that upon encountering a Gram-negative bacterium, the positively charged helical tail of LysSA05 inserts into the outer membrane lipid bilayer, creating localized disruptions or pores. This perturbation of the outer membrane may enable the ~18 kDa catalytic domain to translocate, or at minimum, access, the underlying PG layer, which it can then enzymatically degrade. In essence, LysSA05 harbors its own intrinsic AMP-like module that functions similarly to an OMP. This built-in mechanism explains LysSA05’s ability to exert direct lytic activity against Gram-negative bacteria without the need for external helper agents, a unique and valuable feature that distinguishes it from most known endolysins.

LysSA05 belongs to a rare class of phage-derived endolysins with intrinsic activity against Gram-negative bacteria. Notably, endolysins such as gp279 from *Pseudomonas* phage OBP and LysAB2 from an *Acinetobacter baumannii* phage harbor C-terminal amphipathic helices that allow them to breach the outer membrane, thereby conferring anti-Gram-negative activity (Cornelissen et al., 2012; Lai et al., 2011). Similarly, gp144 from *Pseudomonas aeruginosa* phage φKZ and ABgp46 from *A. baumannii* phage vb_AbaP_CEB1 have been shown to degrade the cell wall of Gram-negative bacteria and exhibit lytic activity against MDR strains (Paradis-Bleau et al., 2007; Oliveira et al., 2016). Similarly, the endolysin ABgp46 demonstrates broad-spectrum activity against Gram-negative bacteria, including MDR *A. baumannii* (Oliveira et al., 2016). Strikingly, LysSA05 demonstrated potent intrinsic lytic activity against Gram-negative bacteria without the need for external membrane-disrupting agents. Purified LPS degradation assays and antibacterial activity measurements confirmed that this ϵ15-derived endolysin possesses dual functionality: it not only degrades PG through its GH19 catalytic domain but also contains an amphipathic α-helical region at the C-terminus that facilitates outer membrane permeabilization. This dual action enables LysSA05 to overcome the outer membrane barrier, a major obstacle for most native endolysins targeting Gram-negative pathogens. The findings presented here align with previous reports of broad-spectrum or engineered endolysins, such as PlyF307, which also exhibit potent activity against MDR Gram-negative bacteria (Lood et al., 2015). However, the ability of LysSA05 to naturally bypass the outer membrane without auxiliary agents is uncommon and underscores its potential as a highly effective therapeutic enzybiotic.

The dual functional profile of LysSA05 holds considerable promise for combating antibiotic-resistant Gram-negative infections. As a purified endolysin enzyme with both PG-cleaving and membrane-disruptive activities, LysSA05 could potentially be employed as a stand-alone antibacterial agent or as an adjunct to antibiotics and lytic phages, without requiring outer membrane permeabilizers, for therapeutic purposes. Moreover, because it originates from a bacteriophage, LysSA05 may exert lower selective pressure for resistance compared to traditional antibiotics, particularly when used in combination with phages targeting the same bacterial pathogen. However, several challenges remain for the clinical translation of LysSA05. Its stability, half-life, immunogenicity, and potential toxicity must be rigorously evaluated *in vivo* to ensure safety and efficacy. Nonetheless, this study contributes valuable advances in both endolysin characterization and phage purification techniques relevant to therapeutic development. By enriching ϵ15 particles free of endotoxins and cellular debris, this work moves a step closer to making phage therapy safer and more clinically viable. Highly purified phage preparations reduce the risk of inflammatory side effects associated with contaminants, thus supporting their potential for systemic administration. Furthermore, the high-resolution structural insights presented here offer a foundation for engineering hypothetical phage proteins and for deepening our understanding of the molecular mechanisms underlying phage infection. Looking ahead, the properties by LysSA05 provide a compelling framework for the discovery and development of novel phage-derived AMPs and enzybiotics. In summary, this study not only advances our understanding of phage structural biology but also supports ongoing efforts to develop phage-based therapeutics and enzybiotics to address the persistent global threat of MDR bacterial pathogens.

## Data Availability

The datasets presented in this study can be found in online repositories. The complete phage genome sequence of ε15 is available at GenBank under accession number NC_004775.2. Additional datasets, including raw data and analysis files, are provided in the Supplementary Material at: https://www.frontiersin.org/articles/10.3389/fcimb.2025.1643576/full#supplementary-material.
