# Supplementary material for: Expanding structural insights into DNA packaging apparatus and endolysin LysSA05 function of Epsilon15 bacteriophage
Source: Front Cell Infect Microbiol. 2025 Aug 14;15:1643576. doi: 10.3389/fcimb.2025.1643576 (PMC12391199; doi:10.3389/fcimb.2025.1643576)
Supplement: Supplementary file 1 [file DataSheet1.docx]

**Expanding Structural Insights into DNA Packaging Apparatus and Endolysin LysSA05 Function of Epsilon15 Bacteriophage**

**Muhammad Saleem Iqbal Khan^1#^, Ju Wu^1#^,** **Shenlin Ji^1^, Demeng Tan^2^, Bingrui Sui^3^, Shanshan Peng^4^, Jinbiao Zhan^4^, Jiajun Yin^1^*******

^1^Department of General Surgery, Affiliated Zhongshan Hospital of Dalian University, Dalian, Liaoning 116001, China.

^2^Shanghai Public Health Clinical Center, Fudan University, Shanghai 201508, China.

^3^MOE Key Laboratory of Bio-Intelligent Manufacturing, School of Bioengineering, Dalian University of Technology, Dalian, China.

^4^Department of Biochemistry, The Second Affiliated Hospital, School of Medicine, Zhejiang University, Hangzhou 310058, China

^#^Muhammad Saleem Iqbal Khan and Ju Wu contributed equally to this article.

**Address correspondence to** Jiajun Yin, [yinjiajun@dlu.edu.cn](mailto:yinjiajun@dlu.edu.cn)

**Supplementary Figures**

**Supplementary Figures 1:**


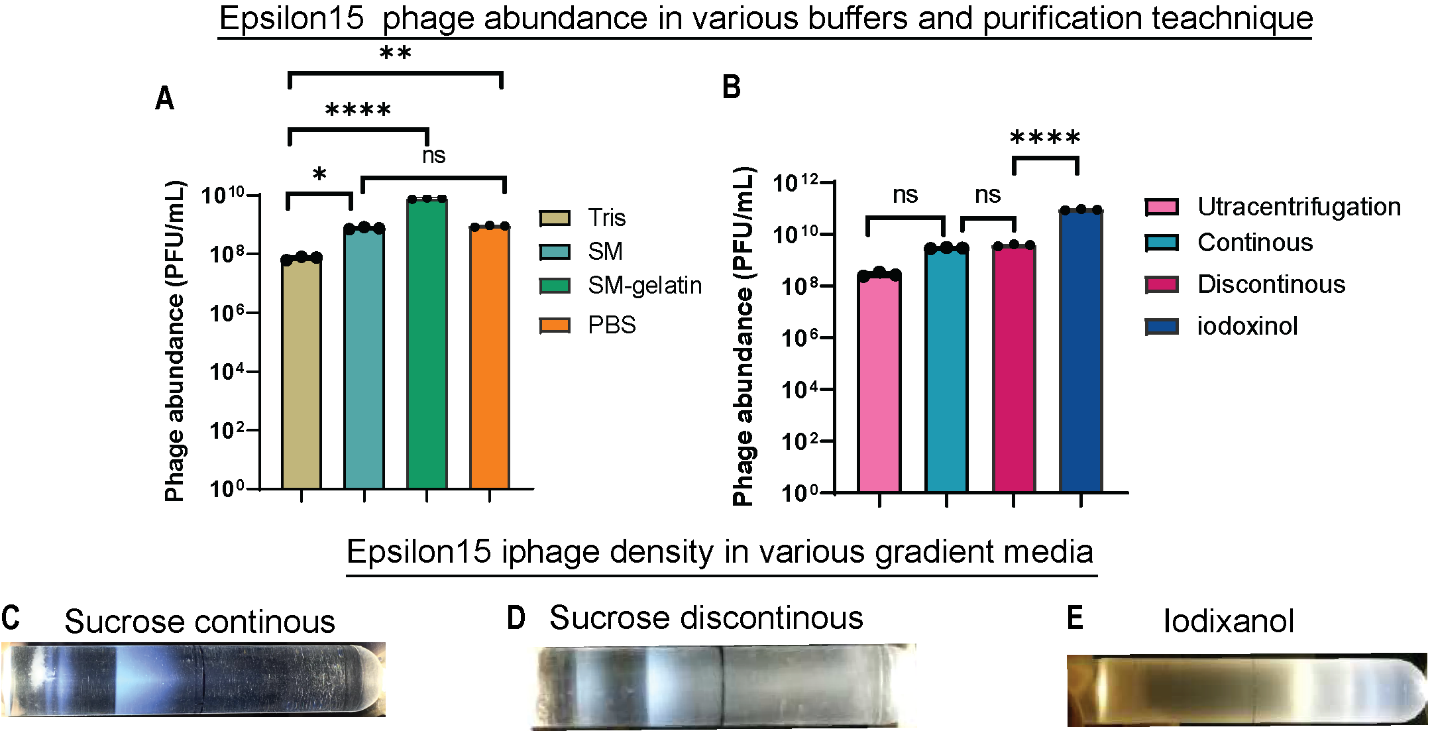


**Supplementary Figures 1.** **Optimization of ε15 phage yield across buffer systems and purification methods.** (A) Phage titers (PFU/mL) of ε15 in different stabilizing buffers: Tris, SM buffer, SM + gelatin, and PBS. (B) Comparison ε15phage titers following various purification protocols. (C–E) Visual comparison of phage band localization in gradient media, with iodixanol showing the most distinct and concentrated band. Data are shown as mean ± SD. Statistical significance is indicated as follows **P* < 0.05, ***P* < 0.01, *****P* < 0.0001; ns = not significant.

**Supplementary Figures 2:**


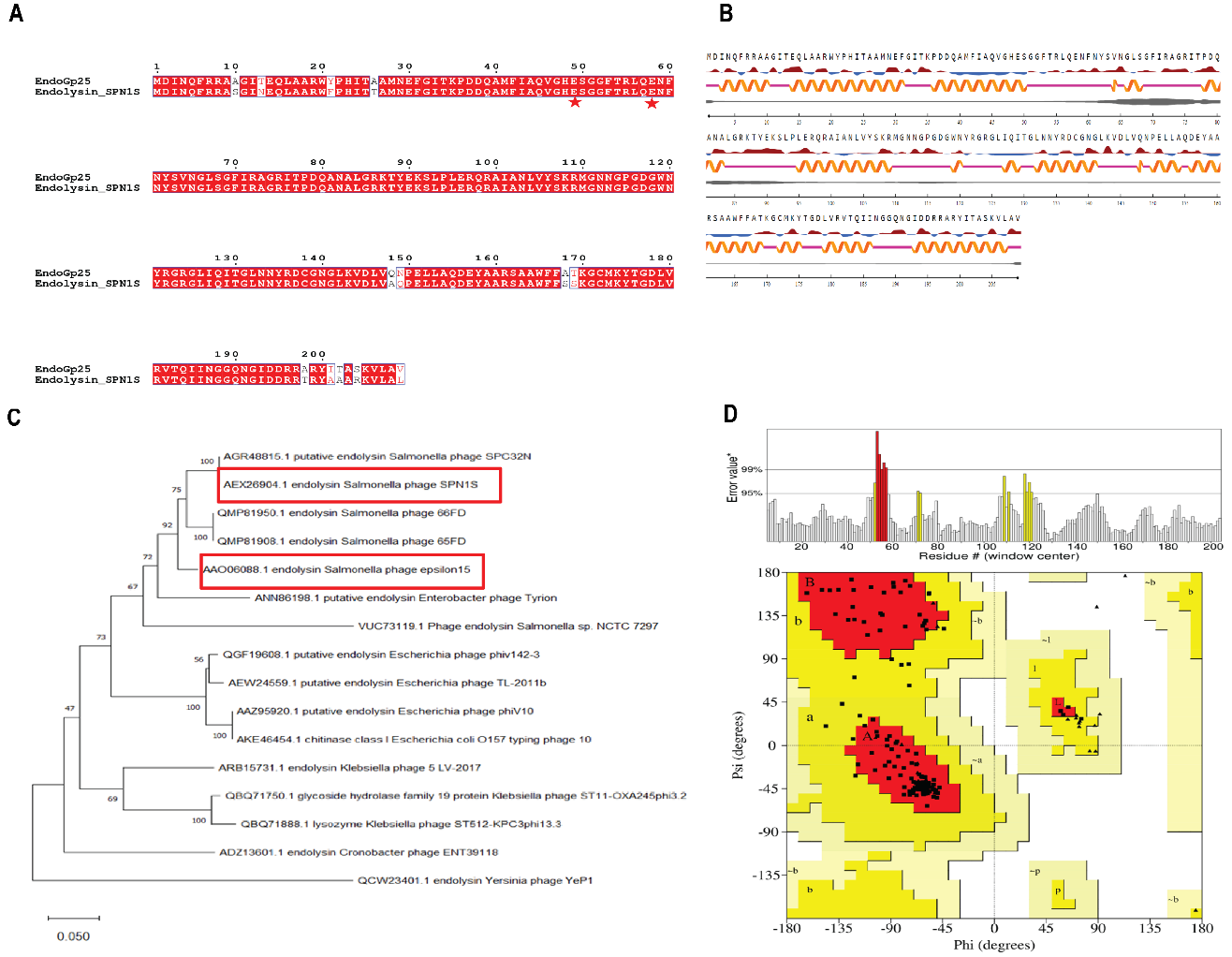


**Supplementary Figures 2**. **Structural and evolutionary analysis of ε15 endolysin LysSA05.** (A) Multiple sequence alignment of LysSA05 with SPN1S endolysin, highlighting conserved residues in red. (B) Predicted secondary structure of LysSA05, showing α-helices (orange), β-sheets (pink), and coil regions (black). (C) Phylogenetic tree of phage endolysins, with LysSA05 clustering closely with SPN1S (highlighted in red) and other *Salmonella* phages. (D) Structural validation of the LysSA05 3D model using the ERRAT and Ramachandran plot analysis, confirming stereochemical reliability.

**Supplementary Figures 3:**


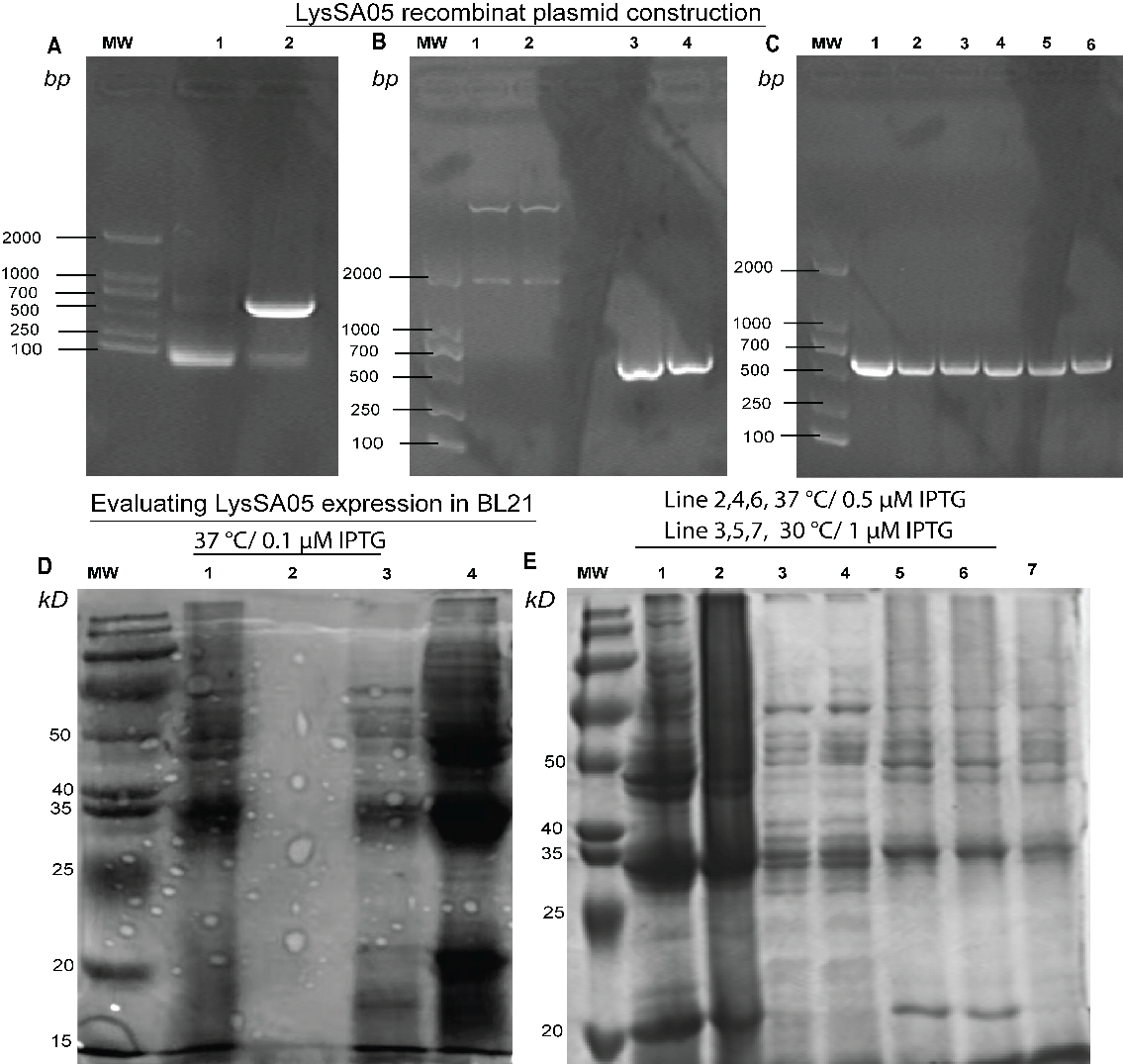


**Supplementary Figures 3.** **Construction and expression of recombinant LysSA05 endolysin.** (A) PCR amplification of the 628 bp *gp05* (*LysSA05*) gene from ε15 phage genomic DNA. (B) Restriction digestion of pET-28a(+) plasmids without insert (lanes 1–2) and with the *gp05* insert (lanes 3–4) using *NcoI* and *XhoI*. (C) Colony PCR screening of recombinant clones confirms successful amplification of *gp05* in all tested colonies (lanes 1–6). (D) SDS-PAGE analysis of protein expression in *E. coli* BL21 induced with 0.1 μM IPTG at 37°C. (E) SDS-PAGE comparison of expression at 37°C with 0.5 μM IPTG (lanes 2, 4, 6) and at 30°C with 1 μM IPTG (lanes 3, 5, 7). A ~25 kDa band indicates successful expression of recombinant LysSA05 (gp05).

**Supplementary Tables**

**Supplementary Table 1:**

**Supplementary Table 1: Bacterial strains, phage, and plasmid used in this study:**

| **Strains, phage, and plasmid** | **Genotype and relevant feature** | **Source** |
| --- | --- | --- |
| *Escherichia coli* DH5α | *E. coli* F- endA1 glnV44 thi-1 recA1 relA1 gyrA96 deoR nupG F80dlacZΔM15 Δ(lacZYA- argF) U169, hsdR17(rKmK+), l | Tiagen |
| *Escherichia coli* Rosetta | DE3 | Novagen |
| *Escherichia coli* BL21 (DH3) | *E. coli* F-ompT hsdS(rB-mB-) gal dcm (DE3) | Tiagen |
| *Salmonella anatum* | serovar Anatum-specific | Gene and antibody Engineering Lab, ZJU |
| *Escherichia coli* C (phix174 host) | *E. coli* (Migula) Castellani and Chalmers ATCC®13706™ | Lot no: 20180215 (biofeng) |
| Expression vector | pET-28 a(+) | Gene and antibody Engineering Lab, ZJU |
| Bacteriophage | Epsilon (ε)-15 | Gene and antibody Engineering Lab, ZJU |

**Supplementary Table 2:**

**Supplementary Table 2: *Salmonella* and *Escherichia* clinical isolates used in this study** Antibiogram: AX: ampicillin; STR: Streptomycin; TET: Tetracycline; CAZ: ceftazidime; ND: not detected, R: Resistant; S: Susceptible; I: intermediate-resistance; all these clinical strains isolated from second affiliated hospitals of Zhejiang university

| ***Salmonella* isolates** | **Antibiogram** | ***E. coli* isolates** | **Antibiogram** |
| --- | --- | --- | --- |
| ZJM-037 | AXR, STRR, TETR, CAZS | ZJM-152 | AXR, STRR, TETS, CAZS |
| ZJM-109 | AXR, STRS, TETR | ZJM-410 | AXR, STRS, TETR, CAZS |
| ZJM-547 | AXR, STRR, TETS, CAZR | ZJM-254 | AXR, STRR, TETR, CAZS |
| ZJM-126 | ND | ZJM-651 | AXR, STRR, TETR, CAZS |
| ZJM-625 | AXR, STRR, TETS, CAZS | ZJM-312 | AXR, STRR, TETR, CAZS |
| ZJM-077 | AXR, STRR, TETS, CAZS | ZJM-045 | AXR, STRR, TETR, CAZS |
| ZJM-233 | AXS, STRR, TETS, CAZS | ZJM-854 | AXS, STRS, TETR, CAZS |
| ZJM-768 | AXR, STRR, TETR, CAZS | ZJM-948 | AXR, STRR, TETR, CAZS |
| ZJM-431 | AXR, STRR, TETR, CAZI | ZJM-055 | AXR, STRR, TETS, CAZS |
| ZJM-685 | ND | ZJM-576 | AXS, STRR, TETS, CAZS |
| ZJM-324 | AXS, STRS, TETS, CAZS | ZJM-227 | AXR, STRR, TETR, CAZS |
| ZJM-563 | AXR, STRR, TETS, CAZS | ZJM-631 | AXR, STRR, TETS, CAZS |
| ZJM-675 | AXR, STRR, TETR, CAZS | ZJM-478 | AXR, STRS, TETS, CAZS |
| ZJM-871 | AXS, STRR, TETS, CAZS | ZJM-870 | AXR, STRR, TETR, CAZS |
| ZJM-431 | AXR, STRR, TETR, CAZR | ZJM-536 | AXS, STRR, TETS, CAZS |
| ZJM-256 | AXR, STRR, TETS, CAZS | ZJM-798 | AXR, STRR, TETR, CAZI |
| ZJM-299 | AXR, STRR, TETS, CAZR | ZJM-367 | AXRR, STRR, TETS, CAZR |
| ZJM-410 | ND | ZJM-231 | AXS, STRR, TETR, CAZS |
| ZJM-349 | ND | ZJM-459 | AXS, STRR, TETS, CAZS |
| ZJM-481 | AXS, STRR, TETR, CAZS | ZJM-096 | AXR, STRR, TETS, CAZR |
